# Isonitrosoacetophenone Drives Transcriptional Reprogramming in *Nicotiana tabacum* Cells in Support of Innate Immunity and Defense

**DOI:** 10.1371/journal.pone.0117377

**Published:** 2015-02-06

**Authors:** Arnaud T. Djami-Tchatchou, Mmapula P. Maake, Lizelle A. Piater, Ian A. Dubery

**Affiliations:** Department of Biochemistry, University of Johannesburg, Auckland Park, South Africa; National Institute of Plant Genome Research, INDIA

## Abstract

Plants respond to various stress stimuli by activating broad-spectrum defense responses both locally as well as systemically. As such, identification of expressed genes represents an important step towards understanding inducible defense responses and assists in designing appropriate intervention strategies for disease management. Genes differentially expressed in tobacco cell suspensions following elicitation with isonitrosoacetophenone (INAP) were identified using mRNA differential display and pyro-sequencing. Sequencing data produced 14579 reads, which resulted in 198 contigs and 1758 singletons. Following BLAST analyses, several inducible plant defense genes of interest were identified and classified into functional categories including signal transduction, transcription activation, transcription and protein synthesis, protein degradation and ubiquitination, stress-responsive, defense-related, metabolism and energy, regulation, transportation, cytoskeleton and cell wall-related. Quantitative PCR was used to investigate the expression of 17 selected target genes within these categories. Results indicate that INAP has a sensitising or priming effect through activation of salicylic acid-, jasmonic acid- and ethylene pathways that result in an altered transcriptome, with the expression of genes involved in perception of pathogens and associated cellular re-programming in support of defense. Furthermore, infection assays with the pathogen *Pseudomonas syringae* pv. *tabaci* confirmed the establishment of a functional anti-microbial environment *in planta*.

## Introduction

Due to the lack of a circulative adaptive immune system, plants have adapted to biotic stressors by developing resistance mechanisms to recognize and counter-attack prospective colonists and pathogens [1]. Resistance involves several pre-formed and inducible mechanisms that inhibit pathogen growth and can be dependent on host resistance- as well as pathogen avirulent genes [2]. Plant resistance is considered effective if it remains operative even in an environment which favors contagious diseases. Resistance, supported by the innate immune system of plants, can be induced by elicitors of biotic or abiotic origin. Induced-resistance (IR) has shown much promise as a possible solution for plant protection and in the development of novel crop protection strategies [3].

Certain naturally occurring or synthetic organic and inorganic chemicals (‘plant activators’) have been reported to induce resistance in plants [4]. These include salicylic acid (SA) and its structural or functional analogs 2,6-dichloroisonicotinic acid (INA) and its methyl ester and benzo-(1,2,3)-thiadiazole-7-carbothioic acid S-methyl ester (BTH), all of which are known to be highly potent activators of systemic acquired immunity (SAR). Others not related to SA include β-aminobutyric acid (BABA), riboflavin, saccharin, hexanoic acid, azelaic acid, pipecolic acid and sulphonamides [4]. Interventions to prime plant defenses could act as valuable tools for crop protection. However, the biochemical action mechanism(s) of these diverse chemicals are not fully understood and more studies are needed to optimise their plant protective- and beneficial effects.

The current study was conducted by using isonitrosoacetophenone (INAP), a novel compound that was originally isolated in a prenylated form from citrus peel tissue undergoing oxidative stress [5], as chemical inducer of plant defense. Previously, metabolomic analyses of INAP-treated tobacco cells identified response-associated metabolites known in the context of plant stress- and defense responses. These include benzoic- or cinnamic acid as well as flavonoid derivatives. INAP thus affects the shikimate-, phenylpropanoid- and flavonoid pathways [6]. Here, the transcriptome status of INAP-treated *Nicotiana tabacum* cells was investigated using Annealing Control Primer (ACP)-based differential display reverse transcription polymerase chain reaction (DDRT-PCR) in combination with 454 pyro-sequencing and qPCR.

## Materials and Methods

### Plant material and growth conditions

*Nicotiana tabacum* cv Samsun cell cultures were grown at 25°C in the dark in Murashige and Skoog (MS) medium containing 0.25 mg/L 2,4-dichlorophenoxyacetic acid and 0.25 mg/L kinetin (pH 5.8), whilst continuously shaking at 120 rpm [7]. Cells were sub-cultured into fresh medium every 7 d. All of the experiments were conducted 2–3 d after sub-culturing.

### Elicitation and total RNA extraction

Tobacco cell suspensions were treated with 1 mM INAP (Sigma Aldrich, Germany) for 0, 1, 2, 4, 8, 12 and 24 h time points [18]. Non-treated cells served as a negative control. Following elicitation, total RNA was isolated from harvested cells by using the Trizol-reagent method (Invitrogen, Carlsbad, CA, USA). Concentrations were determined using a NanoDrop ND-1000 Spectrophotometer (NanoDrop Inc., Wilmington, DE, USA). The extracted RNA samples were then subjected to DNase treatment using the Promega RQ-1 RNase-free DNase kit (Promega, Madison, WI, USA) according to the manufacturer’s instructions. The samples were further subjected to 2.5 M lithium chloride precipitation [8]. The A_260/280_ and A_260/230_ absorption ratios were determined as quality indexes and RNase inhibitor (Rnasin Ribonuclease inhibitor, Promega) was added immediately after quantification. The purified mRNA samples were aliquoted and stored at -20°C for later use. The RNA integrity of all samples were examined by electrophoresis on an 1.5% agarose gel in 1X Tris-Borate-EDTA (TBE) buffer containing 0.5 μg/mL ethidium bromide. The gels were visualized under UV light using a Bio-Rad Image Analyzer and Quantity One Version 4.6.1 Software (Bio-Rad Laboratories, Johannesburg, South Africa).

### Differential display mRNA profiling

Annealing control primer-differential display reverse transcription—polymerase chain reaction (ACP-DDRT-PCR) was conducted as a two-step reaction, with the reverse transcription using Moloney Murine Leukemia Virus reverse transcriptase enzyme (MMLV-RT, Promega) and PCR amplification using GeneFishing DEG premix 101–104 kits according to the manufacturer’s (Seegene Inc., Seoul, South Korea) instructions. The sequences of the arbitrary ACP primers, dT-ACP1 and dT-ACP2 are reported in S1 Table. ACP-PCR amplicons were evaluated on 1.5% agarose gels stained with Gel Green stain (Biotum Inc., Hayward, CA, USA). Amplicons originating from INAP-treated samples were analyzed in parallel to the control (0 h) amplicons in order to identify differential expressed bands. Bands of up-regulated genes were excised from the gel with sterile scapel blades and DNA extracted using the Zymoclean Gel DNA Recovery kit (Zymo Research, Irvine, CA, USA). The extracted DNA was re-amplified using the GoTaq Flexi DNA polymerase kit (Promega) and universal primers with sequences complementary to the 5´ end of the ACP-primers (S1 Table).

### Pyro-sequencing and bioinformatics analyses

Prior to sequencing, the quality and quantity assessment of amplicons was done using the Bioanalyzer (Agilent, Santa Clara, CA, USA) and fluorometer by Inqaba Biotec, Pretoria, South Africa. Approximately 2 μg (at a concentration of at least 50 ng/μL) of purified DNA samples were sequenced on a GS-FLX sequencer using the 454 high-throughput pyro-sequencing technology (Roche Diagnostics, Mannheim, Germany) by Inqaba Biotec, Pretoria, South Africa. After sequencing, the data from the 454-read sequences of each sample were assembled into contigs using the proprietary Roche 454 Newbler Assembler software. Not all reads were assembled into contigs for each sample set and these are indicated as singletons. Subsequently, the sequences were annotated using the Basic Local Alignment Search Tool (BLAST) from the National Center for Biotechnology Information (NCBI, www.ncbi.nlm.nih.gov). Similarities at the nucleotide level were identified using BLASTN and protein homologies were identified using the non-redundant protein databases BLASTX [9]. Each gene was then placed into a functional category based on the putative function thereof.

### Quantitative RT-PCR reaction analyses

qRT-PCR was performed to validate the results of the differential display analysis using the Rotor Gene-3000A instrument (Corbett Research, Qiagen, Hamburg, Germany). Based on their putative function in plant defense and according to their identification revealed by sequence analysis, seventeen genes, representing members of the functional categories identified, were selected for gene expression analysis. Additional time points of 12 h and 24 h were introduced in order to detect transient expression of the genes. The selected gene transcripts were β-1,3-Glucanase (HSZW1U101BMRIS), Cysteine proteinase (Contig00026), Cyclophilin (Contig00001), Ethylene response element-binding protein (EREBP—Contig00040), Thioredoxin (Contig00045), Heat shock protein 90 (HSP90—Contig00048), Small Sar1 GTPase (SAR1-GTPase—Contig00050), Chitinase (gi|62719020:1–272), Avr9/Cf-9 rapidly elicited (ACRE-261—Contig00093), Biotic cell death-associated protein (BIOTHSZW1U101A3L23), Pheophorbide oxygenase A (HSZW1U101A9XP5), SGT1 (gb|AF516180.1|), NPR1 (gb|AF480488.1|), RAR1 (gb|AF480487.1|), Cytochrome P450 (HSZW1U101BQZCB), PR-1a (gb|JN247448.1|) and PR-1b (gb|X66942.1|).

Primer pairs were designed using the Integrated DNA Technologies (IDT)’s PrimerQuest tool (http://eu.idtdna.com/Scitools/Applications/Primerquest/Default.aspx) from the sequences obtained and Genbank (www.ncbi.nlm.nih.gov/genbank). All primer sequences are shown in S2 Table. Total RNA was reverse transcribed to cDNA using ImPromII RT enzyme (Promega) according to manufacturer’s instructions. The resulting cDNA was then used for qPCR amplification using the Sensimix dT kit (Quantace, London, UK) according to the supplied instructions. The PCR cycling conditions were as follows: 95°C for 10 min, followed by 40 cycles of amplification at 95°C for 10 sec, 60°C for 15 sec, and 72°C for 12 sec. Three biological repeats were used with two technical repeats of each.

The relative standard curve method [10] was used to quantify the selected genes and the data normalized using two reference genes; *elongation factor 1-alpha (Elf α)* and *18SrRNA* [11]. Data sets were statistically compared between non-treated controls and treated samples at each time point using one-way analysis of variation (ANOVA) with the statistical analysis software GraphPad inStat 3 (GraphPad software, San Diego, CA, USA). The confidence level of all analyses was set at 95%, and values with P < 0.05 were considered significant.

### *In planta* growth assays

*Pseudomonas syringae* pv. *tabaci* 6605 was obtained from Prof. Y. Ichinose (Okayama University, Okayama, Japan) and maintained as described [12]. Overnight cultures grown in King’s B medium were diluted in 10 mM MgSO_4_ to an OD_600_ of 0.002 (equivalent to 1.10^6^ cfu/ml). Tobacco plants (*Nicotiana tabacum* ‘Xanthi NC’) were grown at 25°C with a 16 h photoperiod. Detached leaves were placed in a solution of 0.5 mM INAP dissolved in 10 mM MgSO_4_ (treatment) or 10 mM MgSO_4_ (controls). After 24 h of conditioning, leaf disks of 10 mm diameter were punched from treated and control leaves, and rinsed in sterile distilled water. Leaf discs were vacuum infiltrated with a suspension of bacteria (OD_600_ = 0.002 / 1 x 10^6^ cfu/ml) in 10 mM MgSO_4_ using a Buchner flask, and placed on wetted filter paper in Petri dishes at 25°C under a 12 h light/ 12 h dark cycle. At 2 and 4 d post-inoculation, leaf disks were rinsed in 15% H_2_O_2_ to sterilize the leaf surface and washed with sterile dH_2_O. Three discs were homogenized (using a hand held pestle in an Eppendorf tube) in 200 μl sterile dH_2_O followed by vortexing and brief centrifugation. One hundred μl of the suspension was added to 900 μl sterile dH_2_O and serially diluted. Dilutions of 10^4^—and 10^5^ -fold (100 μl each) were spread on KB plates and the numbers of colonies growing after 48 h incubation at 27°C were calculated [12].

## Results and Discussion

This study aimed to assess changes in gene expression associated with isonitrosoacetophenone-associated defense induction in *Nicotiana tabacum*. mRNA differential display was successfully used for detection and recovery of up-regulated PCR amplicons as also previously used to identify and isolate genes involved in plant innate immunity [7,13]. The different time points (1–24 h post treatment) were selected to be able to detect both ‘early response’ (1–4 h) gene transcripts, associated with signalling events, as well as ‘late response’ transcripts (8–24 h), associated with an activated defense response.

### Identification of differentially expressed gene transcripts

**mRNA differential display**. Total RNA was isolated from tobacco cells following elicitation with 1 mM INAP for 0, 1, 2, 4, 8, 12 and 24 h. RNA integrity was confirmed by the sharpness of distinct 28S and 18S rRNA bands visualized by electrophoresis. Based on the ratios obtained for A_260/280_ and A_260/230_, as well as the integrity, it was concluded that the RNA was of high quality and free from protein, polyphenol and polysaccharide contamination. The isolated mRNA was reverse transcribed and amplified using ACP differential display technology. The ACP-DDRT-PCR products (amplicons) were separated on a 1.5% agarose gel. The experimental samples were run in parallel to the non-treated controls in order to identify differentially expressed genes (DEGs). Various genes, as represented by the amplicons, were differentially expressed at the time points investigated (0, 1, 2 and 4 h) (S1a Fig.).

**Re-amplification of ACP-DDRT-PCR products**. cDNA extracted from the excised amplicon bands was re-amplified using universal primers with sequences homologous to the universal portion of the ACP primers. The re-amplified PCR products were evaluated on a 1.5% agarose gel to verify that only a single product was obtained (S1b Fig.).

**Fig 1:**
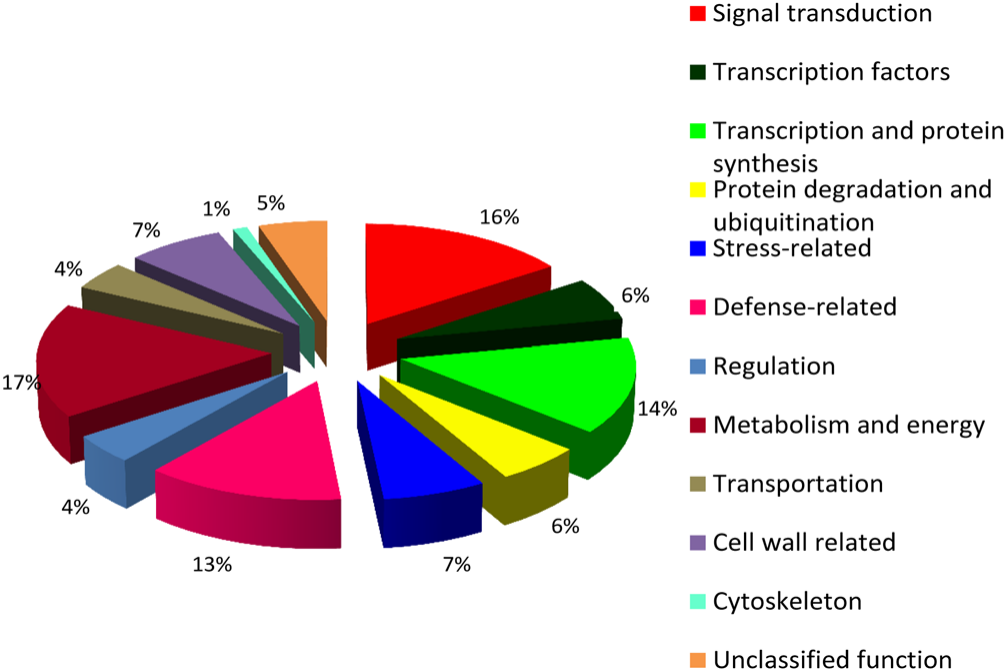
Classification of INAP-induced transcripts according to functional categories. Pie-chart showing genes induced by INAP treatment in cultured tobacco cells, identified through ACP-DDRT-PCR, pyro-sequencing and BLAST analyses, as expressed in percentage values.

**Sequencing, bioinformatics analyses and gene identification**. Pyro-sequencing technology constitutes a powerful tool for investigating transcriptome changes and gene discovery of non-model species such as tobacco [13–15]. Following 454 pyro-sequencing of the re-amplified DDRT-PCR amplicons, 14579 reads were generated that resulted in 198 contigs after the assembly. A further 1758 reads were produced that did not overlap with other reads or could not be assembled, and labelled as singletons. Subsequently, the sequences were annotated using both BLASTN to identify similarities at nucleotide level and BLASTX to identify similarities at protein level. Some of the sequences showed similarities with genes in both BLASTN and BLASTX, while others showed similarities in either one of the programs. Each provisionally identified gene transcript was further classified into a functional group based on the putative role (Table 1, S3 Table). We identified several genes of interest associated with signaling, priming and defense; and classified these under signal perception and -transduction, response regulation, transcription activation, protein ubiquitination, protein synthesis and folding, vesicles and transport, stress-responsive, defense-related, metabolism and energy, cytoskeleton and cell wall-related, respectively (Fig. 1). Some of the identified genes may also fit into more than one single functional category.

**Table 1:** Summary of selected genes differentially expressed in tobacco cell suspensions in response to INAP treatment.

SIMILAR SEQUENCE FROM BLAST DATABASE	E-value	Max identity %
SIGNAL PERCEPTION AND TRANSDUCTION		
*Nicotiana attenuata* lectin-domain receptor-like kinase (lecRK1) mRNA, lecRK1.1 allele, complete cds	7.00E-16	78
*Arabidopsis thaliana* leucine-rich repeat receptor-like protein kinase	5.00E-04	92
*Glycine max* NB-LRR type disease resistance protein Rps1-k-1 (Rps1-k-1) genes, complete cds	0.006	80
*Arabidopsis thaliana* putative calcium-binding protein CML13 (AT1G12310) mRNA	9.00E-19	75
*Pinus sylvestris* mRNA for CuZn superoxide dismutase, clone PS3	7.00E-05	85
*Nicotiana tabacum* MAPK gene for wound induced protein kinase (WIPK), complete cds	2.00E-12	80
*Nicotiana tabacum* mRNA for 14–3–3-like protein	2.00E-08	85
*Nicotiana attenuata* JAR1-like protein (JAR6) mRNA, complete cds	0.084	93
*Oryza sativa* a8 gene for plasma membrane H+-ATPase	0.33	100
*Solanum lycopersicum* protein phosphatase 2C (DIG3), mRNA	9.00E-38	91
*Nicotiana tabacum* mRNA for GTP-binding protein, GTPase SAR1	4.00E-123	100
*Nicotiana benthamiana* Rab GDP dissociation inhibitor (GDI) mRNA, complete cds	2.00E-101	98
*Nicotiana tabacum* Rho GDP dissociation inhibitor (GDI) mRNA, complete cds	6.00E-145	91
RESPONSE REGULATION		
*Nicotiana attenuata* SGT1 mRNA, complete cds	2.00E-09	98
*Nicotiana tabacum* NtARL8b mRNA for ADP-ribosylation factor-like 8b, complete cds	8.00E-42	100
*Nicotiana tabacum* Bax inhibitor 1 (BI-1) mRNA, complete cds	8.00E-87	99
*Petunia x hybrida* PhRR1 mRNA for type-A response regulator, complete cds	1.00E-22	87
*Populus trichocarpa* NPR1/NIM1-like regulatory protein, mRNA	0.18	84
*Solanum lycopersicum* ripening regulated protein DDTFR19, mRNA	5.00E-13	91
TRANSCRIPTION		
*Nicotiana tabacum* DNA-directed RNA polymerase IIb (NT193) mRNA, complete cds	5.00E-110	96
*Nicotiana tabacum* mRNA for TATA binding protein (TBP), complete cds	1.00E-90	98
*Arabidopsis thaliana* putative transcription factor MYB108 mRNA, complete cds	5.00E-06	92
*Arabidopsis thaliana* bZIP-like transcription factor protein	0.002	52
*Capsicum annuum* putative ethylene-responsive element binding protein (JERF1) mRNA	6.00E-96	87
*Malus x domestica* NAC domain class transcription factor	0.002	71
*Nicotiana tabacum* mRNA for WRKY transcription factor NtEIG-D48, complete cds	2.00E-07	97
*Juglans regia* mRNA for putative Cys2-His2 zinc finger transcription factor (zfp2 gene)	2.00E-08	76
*Vitis vinifera* transcription factor APETALA2 (AP2) mRNA, complete cds	9.00E-05	85
PROTEIN UBIQUITINATION AND DEGRADATION		
*Vitis vinifera* NEDD8-activating enzyme E1 catalytic subunit-like (LOC100256207), mRNA	4.00E-18	87
*Pyrus pyrifolia* var. *culta* mRNA for putative E3 ubiquitin ligase, partial cds	3.00E-09	100
*Capsicum annuum* ubiquitin-conjugating protein mRNA, complete cds	2.00E-12	77
*Solanum nigrum* clone 110 ubiquitin extension protein (Ubi2) mRNA, complete cds	1.00E-16	96
*Ricinus communis* 26S protease regulatory subunit 6b, putative, mRNA	5.00E-12	96
*Ricinus communis* proteasome subunit beta type 5,8, putative, mRNA	4.00E-20	82
PROTEIN SYNTHESIS AND FOLDING		
*Nicotiana tabacum* putative RNA binding protein (QRRBP-1) mRNA, partial cds	3.00E-105	93
*Nicotiana tabacum* clone 7 poly(A)-binding protein (PABP) mRNA, partial cds	3.00E-151	98
*Nicotiana tabacum* EF-1-alpha-related GTP-binding protein (SUP1) mRNA, complete cds	4.00E-144	96
*Nicotiana tabacum* cyclophilin-like (CYP1) mRNA, complete sequence	0	97
VESICLES AND TRANSPORTATION		
*Glycine max* ABC transporter B family member 25-like (LOC100810510), mRNA	2.00E-15	85
*Nicotiana tabacum* NtMATE2 mRNA for multi antimicrobial extrusion family protein	1.00E-149	98
*Solanum tuberosum* clone 021G11 translocon-associated protein (TRAP) beta family protein	2.00E-81	93
*Pyrus pyrifolia* var. *culta* mRNA for putative tonoplast intrinsic protein (TIP) 1, partial cds	7.00E-04	100
*Arabidopsis thaliana* Golgi SNARE 12 protein	4.00E-04	34
*Vitis vinifera* ER-Golgi intermediate compartment protein 3-like (LOC100267365)	9.00E-04	83
*Arabidopsis thaliana* endoplasmic reticulum vesicle transporter protein	5.00E-10	86
*Nicotiana plumbaginifolia* mRNA for calreticulin	9.00E-19	95
STRESS-RELATED RESPONSES		
*Nicotiana tabacum* NtHsp90 mRNA for heat shock protein 90, complete cds	4.00E-91	90
*Nicotiana tabacum* mRNA for putative glutathione S transferase (GST1 gene), clone EBR-52	1.00E-57	87
*Nicotiana tabacum* mRNA for glutathione S-transferase, complete cds	2.00E-53	98
*Arabidopsis thaliana* universal stress protein (USP) family protein (AT3G53990) mRNA	5.00E-31	79
*Nicotiana tabacum* cytosolic class I small heat shock protein 3A (sHSP3A) gene, promoter region and complete cds	9.00E-98	99
*Nicotiana tabacum* mRNA for thioredoxin peroxidase	1.00E-104	100
*Vitis vinifera* serine hydroxymethyltransferase, mitochondrial-like (LOC100245411), mRNA	7.00E-15	87
*Solanum commersonii* stress responsive cyclophilin (SCCYP1) mRNA	3.00E-05	97
DEFENSE RESPONSES		
*Nicotiana tabacum* Avr9/Cf-9 rapidly elicited protein 261 (ACRE261) mRNA, partial cds	3.00E-40	83
*Nicotiana glutinosa* biotic cell death-associated protein (CDM1) mRNA, complete	8.00E-37	75
*Nicotiana tabacum* basic β-1,3-glucanase {clone FB7–5(1)}	1.00E-15	96
*Elaeis guineensis* class II chitinase (CHI2) mRNA, partial cds	3.00E-06	92
*Nicotiana tabacum* partial mRNA for putative stress related chitinase (cht STR1 gene), clone CHO3E10	1.00E-117	98
*Nicotiana tabacum* mRNA for pre-pro-cysteine proteinase	8.00E-159	98
*Arabidopsis thaliana* defensin-like protein 226	2.00E-04	44
*Nicotiana tabacum* pheophorbide A oxygenase 1 mRNA, complete	4.00E-109	99
*Nicotiana tabacum* osmotin pathogenesis-related protein homolog	4.00E-07	100
*Nicotiana tabacum* NtLTP3 gene for lipid transfer protein, complete cds	2.00E-41	93
METABOLISM AND ENERGY		
*Nicotiana tabacum* TCP1 mRNA for cytochrome P450, complete cds	4.00E-94	99
*Betula luminifera* 4-coumarate coenzyme A ligase (4CL) mRNA, complete cds	0.003	89
*Pyrus pyrifolia* var. *culta* mRNA for putative caffeoyl-CoA O-methyltransferase, partial cds	1.00E-05	92
*Rubus coreanus* cinnamate-4-hydroxylase mRNA, complete cds	8.00E-08	87
*Petunia x hybrid* flavin monoxygenase (FMO)-like protein mRNA, fzy-R27 allele, complete cds	0.081	81
*Nicotiana tabacum* mRNA for 1-aminocyclopropane-1-carboxylate oxidase	3.00E-47	81
*Gardenia jasminoides* GjUGT4 mRNA for UDP-glucose glucosyltransferase, complete cds	7.00E-04	100
*Nicotiana tabacum* NtGT2 mRNA for glucosyltransferase, complete cds	2.00E-51	94
*Medicago truncatula* cytochrome c oxidase subunit (MTR_1g006950)	1.00E-16	98
*Solanum tuberosum* mRNA for NADH-ubiquinone oxidoreductase subunit	1.00E-23	78
CELL WALL-RELATED AND CYTOSKELETON		
*Nicotiana sylvestris* mRNA for glycine rich protein 2 (GRP2)	5.00E-161	98
*Nicotiana tabacum* mRNA for proline-rich protein EIG-I30, complete cds	1.00E-178	100
*Glycine max* cellulose synthase-like protein H1-like	1.00E-05	76
*Nicotiana tabacum* partial mRNA for pectin methylesterase	3.00E-34	81
*Nicotiana glauca* mRNA for putative arabinogalactan-protein precursor	3.00E-47	82
*Pyrus pyrifolia* var. *culta* mRNA for putative annexin, partial cds	2.00E-04	94
*Nicotiana tabacum* gene for extensin, complete cds	2.00E-73	100
*Nicotiana tabacum* mRNA for alpha-tubulin (tubA2 gene)	4.00E-36	100

Accession numbers of similar sequences are given in S3 Table.

**Fig 2:**
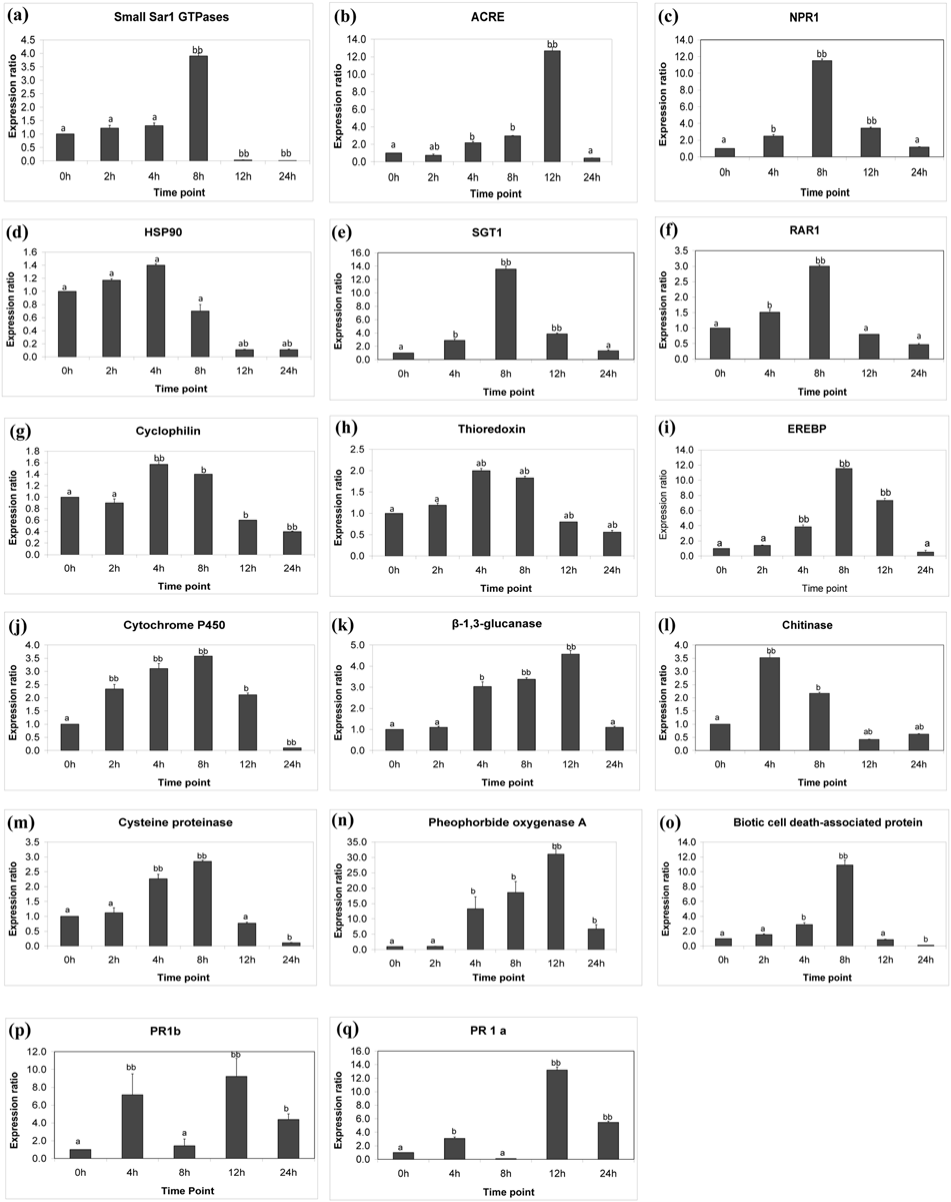
qPCR analysis of differential gene expression kinetics in *Nicotiana tabacum* cells following induction with INAP. The data was normalized using *Elf α* and *18S* to give the relative gene expression wherein error bars represent the standard error of mean. Expression analysis was performed on three biological repeats with two technical repeats of each. (a) SAR1-GTPase, (b) Avr9/Cf-9 rapidly elicited (ACRE-261), (c) NPR1, (d) Heat shock protein 90 (HSP90), (e) SGT1, (f) RAR1, (g) Cyclophilin, (h) Thioredoxin, (i) Ethylene response element binding protein (EREBP), (j) Cytochrome P450, (k) β-1,3-Glucanase, (l) Chitinase, (m) Pre-pro-cysteine proteinase, (n) Pheophorbide oxygenase A, (o) Biotic cell death-associated protein, (p) Pathogenesis-related protein-1b and (q) Pathogenesis-related protein-1a. Error bars: (a) indicates no significant differences, with P > 0.05, (ab) indicates a significant difference with P < 0.05, (b) indicates a highly significant difference with P< 0.01 and (bb) indicates a highly significant difference with P< 0.001.

**Fig 3:**
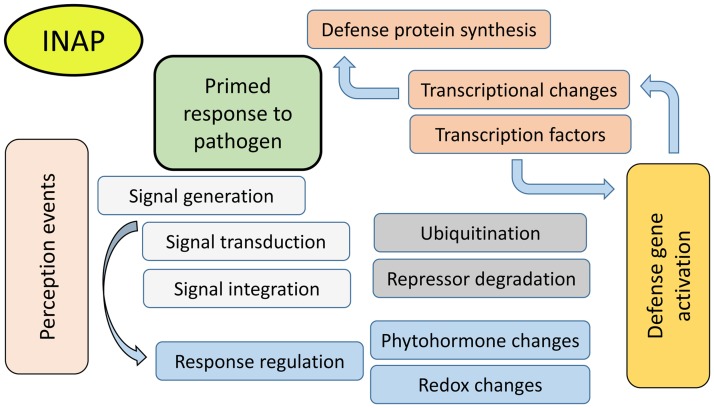
Schematic diagram illustrating the priming action of INAP on *N. tabacum* cells. Results show a broad activation of cellular responses involved in innate immunity from pathogen or PAMP perception to the eventual enhanced innate immune response. Details of the gene transcripts found to be up-regulated in response to treatment of cultured cells by INAP are given in the main text.

**Gene expression analysis**. Quantitative expression analysis of seventeen genes was performed to validate whether the transcript-derived sequencing data reflected the gene expression. This was normalized against *Elf α* and *18S rRNA* to give the relative gene expression wherein error bars represent the standard error of mean (SEM) (Fig. 2a-q). The results indicated that the changes to the transcriptome were dynamic, with transcripts exhibiting different expression kinetics that can be described as early- (2–4 h), mid- (8 h) and late- (>12 h) responses. The fold expression varied from relatively low (>2 fold) to high (>20 fold) compared to the basal levels of non-treated cells. Expression was transient and following maximum activation at 4, 8 or 12 h, transcript levels decreased but (i) remained relatively high (PR1a, PR1b and Pheophorbide oxygenase A), (ii) returned to basal levels (β-1,3-Glucanase, Chitinase, ACRE, NPR1, SGT1, RAR1, EREBP) or (iii) even below the levels initially determined in non-treated control cells (Small SAR1 GTPase, HSP90, Cyclophilin, Cytochrome P450, Cysteine proteinase, Thioredoxin and Biotic cell death-associated protein). Results from the combination of the differential display, sequence analyses and the qRT-PCR are discussed below (Fig. 2a-q) and summarized in Fig. 3.

**Signal perception and—transduction**. Plant perception of pathogen attack is associated with networks of signal transduction pathways coupled to transcriptional activation. Lectin domain receptor-like kinases (RLK) and leucine-rich repeat (LRR) receptor-like protein kinases (LRR-RLK) were identified, two of which are listed in Table 1. LRR-RLKs are assumed pattern recognition receptors (PRRs) and known to be inducible receptors for recognition of extracellular pathogen-derived P/MAMPs [16]. Several genes associated with plant signaling events were found to be up-regulated including SAR1-GTPase, calcium-binding protein CML13, jasmonic acid receptor (JAR1)-like protein, mitogen-activated protein kinase (WIPK), ACRE-261 and protein phosphatase 2C (Table 1).

Real time PCR showed that SAR1-GTPase is slightly up-regulated from 2 to 4 h with a maximum expression at 8 h (Fig. 2a). SAR1-GTPases are small monomeric GTP-binding proteins belonging to the Rho subfamily (also known as RACs or ROPs) involved in intracellular signaling pathways downstream of RLKs. Activated RACs/ROPs are capable of receiving a wide variety of inputs and accordingly generate a multitude of specific cellular responses to the stimuli *via* signaling networks involving interacting partners [17]. These include responses supporting innate immunity such as the regulation of reactive oxygen species (ROS) *via* activation of plasma membrane-associated NADPH oxidases [18], the formation of secretory vesicles and vesicle transport between the endoplasmic reticulum (ER) and Golgi apparatus, cytoskeletal dynamics and membrane trafficking and autophagy [17,19] (discussed below).

A related member of the Ras superfamily, a RabG3b GTPase, was reported by [20] as an immunity regulator in Arabidopsis and as an activator of autophagy, which plays a positive role in plant immunity-triggered hypersensitive response (HR) programmed cell death (PCD). Rab- and Rho GDP dissociation inhibitors (GDIs) negatively regulates the GTPases and returns the binary switch to the inactive state, thus ensuring cellular homeostasis. Small G-protein-triggered innate immunity involves the RAR1-SGT1-HSP90 molecular chaperone complex (discussed below).

Expression of genes encoding a calcium-dependent protein kinase (CDPK) and a calcium ion binding protein were up-regulated, indicating Ca^2+^ signaling activities in response to INAP elicitation. Rapid Ca^2+^ influx modulates the activation of elicitor-responsive plasma membrane (PM) H^+^-ATPases that play important roles in plant innate immune responses. These H^+^-ATPases generate a H^+^ gradient across the membrane that energizes many important transport systems in plants. The proton gradient also generates an electrical potential that drives cation uptake through ion channels.

Ca^2+^ activation also serves as a prerequisite for the activation of MAPKs and other defense responses [17]. MAPKs are activated as part of a plant’s early defense responses, often within minutes of pathogen perception and integrate signals from multiple immune sensors [17,21,22]

ACRE (Avr9/Cf-9 rapidly elicited gene) 261 also showed up-regulated expression from 4 h to a significant increase of 12.5 fold at 12 h (Fig. 2b). Upon infection by *Cladosporium fulvum*, tomato *Cf* genes confer resistance through recognition of secreted Avr peptides [23]. Similarly, Cf-9 confer an Avr-dependent HR in tobacco [24]. Many ACRE genes were found to encode regulatory proteins and ACRE-261 was reported as an APK1-like protein kinase [23].

Protein phosphatase 2C (PP2C-type phosphatase) is emerging as an important role player in plant stress signal transduction [25] and was reported as a regulator of defense responses related to SA accumulation and expression of the pathogenesis-related (PR) proteins PR1, PR2, and PR3 in Arabidopsis [26].

**Transcription factor—and response regulators**. Transcription factors (TFs) play a crucial role in the activation and fine-tuning of defense by either regulating specific genes or a cluster of genes [27]. Among the genes found to be up-regulated, some are known to be involved in transcriptional activation (Table 1). Examples of this category are: bZIP-like, EREBP, ERF4, Myb, bHLH and WRKY transcripts, known to be strongly and rapidly expressed in many plant species in response to biotic and abiotic stress factors [28].

Of special interest is the identification of *NPR1* (*non-expressor of PR genes 1*), a key regulatory component that is positioned at the cross-roads of multiple defense pathways. Previous findings showed that NPR1/NIM1-like regulatory protein, a transcriptional cofactor, is an important positive regulator of the SA-dependent signaling pathway and is required for the redox-dependent transduction of the SA signal to modulate expression of antimicrobial and secretory pathway genes needed for the establishment of SAR [29]. In addition, NPR1-dependent SA pathways control the expression of PR-1, β-1,3-glucanase and thaumatin-like genes in Arabidopsis [30]. Other studies revealed that the over-expression of Arabidopsis NPR1 enhanced bacterial and fungal resistance [31]. In this study, in accordance to the mRNA differential display, qPCR showed that the NPR1 was significantly up-regulated from 2 h to 12 h with a 10-fold increase at 8 h (Fig. 2c). Although *NPR1* is constitutively expressed, its transcript levels are responsive following SA treatment or pathogen infection to activate PR-gene expression and SAR. This suggests that the SA signaling pathway is also involved in the tobacco response to INAP elicitation.

**Cytosolic perception-related proteins and associated chaperones**. This study also matched (without qPCR quantification) several putative intracellular disease resistance gene transcripts of the Toll/Interleukin-1 Receptor-Nucleotide Binding Site-Leucine Rich Repeat (TIR-NBS-LRR) type, from non-Solanaceous species, but with low E-score values (refer to S4 Table). *R* genes encoding NBS-LRR proteins are reported to be the largest class of *R* genes isolated from various plant species challenged by pathogen attack [32]. Their activation results in genetic reprogramming and pronounced physiological changes in the infected plant cell, such as the HR, contributing to resistance [33].

Many R proteins require the cytosolic chaperones, HSP90 (Heat Shock Protein 90), SGT1 (Suppressor of the G2 allele of *Skp1*), and RAR1 (Required for *Mla12* Resistance), to form a molecular chaperone complex which is involved in innate immunity and disease resistance-related signaling events [34]. HSP90 plays a major role as a core component for different protein complexes that associate with other co-chaperones and are required for disease resistance against invading pathogens [35]. Similarly, RAR1 is a protein specifically required for plant innate immunity that interacts with HSP90 and SGT1 to maintain proper NB-LRR protein steady-state levels [36]. The up-regulation of HSP90 expression revealed by mRNA differential display was validated by the qPCR which showed that it was slightly up-regulated at 2 and 4 h and down-regulated from 8–24 h post-elicitation (Fig. 2d). Real time PCR revealed that SGT1 was significantly up-regulated at 4, 8 and 12 h with a 12 fold increase at 8 h (Fig. 2e); and RAR1 was similarly up-regulated from 4–8 h followed by a decrease in expression from 12–24 h (Fig. 2f).

These findings implicate the involvement of the HSP90-SGT-RAR complex in the responses triggered by INAP. Based on previous findings and from the qPCR results, the pattern of the expression of these genes seems to be similar to those of the quantified resistance genes [37], indicating that they were indeed required in the regulation of defense-related genes in response to INAP elicitation. It is of interest that these chaperones also act as a core modulator of small G-protein-triggered plant innate immunity [17].

**Protein ubiquitination and -degradation**. During plant-pathogen interactions proteins that negatively regulate plant defense are targeted and degraded for activation of defense responses [38]. The degradation of ubiquitinated proteins is facilitated by the proteasome which is a multi-complex protein system involved in different biological processes including hormone signaling, homeosis and disease resistance [39]. Studies showed that the 26S proteasome and ubiquitin ligase are induced by chitin and that their inhibition suppresses the activation of defense responses [40]. A direct link between disease resistance, ubiquitination and protein degradation is supplied via SGT1 that associates with the SCF (Skp1-Cullin—F-box) ubiquitin ligase complex and the RAR1-SGT1 complex that interacts with the COP9 signalosome, known to regulate ubiquitin-proteasome mediated protein degradation.

Here, transcripts corresponding to an ubiquitin-conjugating, an ubiquitin-extension and protein E3 ubiquitin ligase [39] were identified (Table 1). Ubiquitin ligases regulate the stability and expression of TFs [17] and ubiquitin-proteasome degradation of WRKY TFs and NPR1 has been reported in Arabidopsis [41]. It was proposed that this regulation could play a role in suppressing unnecessary defense activation in the absence of pathogens [42].

**Protein synthesis, protein folding and protein export / secretion**. Protein synthesis is the main core for the success of plant defense. Following reprogramming of the transcriptome, new protein synthesis is needed as part of the ‘defensome’ in order for the plant to launch an effective and timeous defense response [43]. Following translation on membrane-bound ribosomes, translocation into the endoplasmic reticulum occurs where proteins are subjected to chaperone-assisted folding and assembly. Expression of several genes in the functional category ‘protein synthesis’ were up-regulated, amongst which: an RNA-binding protein, poly(A)-binding protein, EF-1-alpha-related GTP-binding protein (Table 1) and other proteins involved in ribosome structure and function (S4 Table). RNA-binding proteins can package, organise and protect RNA, and prepare it for post-transcriptional processes [44]. RNA-binding proteins are induced in plants due to environmental stress [45] as well as elicitation by lipopolysaccharides (LPS) [46].

Cyclophilins are involved in protein folding as peptidyl-prolyl *cis-trans* isomerases and facilitate the folding of newly synthesised proteins, and the re-folding of proteins damaged during plant defense [47]. Cyclophilins have also been postulated to act as cellular receptors, play a role in protein transport across membranes and intracellular trafficking, triggering of membrane channels, the formation of multisubunit protein complexes [48], mRNA processing, protein degradation and signal transduction [47]. Even though cyclophilins are known to be ubiquitous and constitutively expressed, up-regulation can occur due to stress responses [49], e.g. the accumulation of potato cyclophilin in response to SA and *Phytophtora infestans* [50].

The expression pattern of cyclophilin shown by qPCR (Fig. 2g) is similar to those of the defense-related genes up-regulated mostly at 4 h and 8 h, indicating a strong correlation of gene expression in the response of the cells to INAP elicitation. A transcript for thioredoxin was another early response significantly up-regulated at 4 h and 8 h (Fig. 2h). Thioredoxins function in redox signaling affecting NPR1 and are also involved in protein folding where they act as chaperones and isomerases of disulphides in protein folding [51].

Calreticulin was also identified as an INAP-responsive transcript. The protein is an endoplasmic reticulum-resident, calcium-binding molecular chaperone, functioning in regulating SA levels and important in regulating plant defense against pathogens [52].

Execution of the immune response relies in part on the exocytosis pathway leading to vesicle-associated and SNARE (soluble N-ethylmaleimide sensitive factor attachment protein receptors) protein-mediated secretion of defense-related proteins [53]. SNARE proteins are also involved in mediating vesicle fusion through exocytosis [54]. Here, the identification of Golgi SNARE-12 protein, the ER-Golgi intermediate compartment protein and the ER vesicle transporter protein (Table 1) points towards activation of these processes.

**Phytohormone-related responses**. Crosstalk between different hormone systems allows the plant to respond appropriately to a particular mode of pathogen infection and to integrate biotic and abiotic stimuli [55]. The activation of an ethylene response factor (ERF) and jasmonic acid receptor (JAR1) suggest that both ethylene (ET) and jasmonic acid (JA) may serve as signaling molecules in the response of tobacco cells to INAP. ET and JA are involved in activation of different plant defense responses, especially in the induced systemic resistance (ISR) signaling pathway along with NPR1 [41,56], which was up-regulated from 4 to 12 h (Fig. 2c).

Up-regulation was also observed for EREBP known to be responsible in part for mediating the response to ET by binding to the promotors of target genes and activating downstream ET responses [57,58]. In addition, during plant response to pathogens, ET biosynthesis increases rapidly and subsequently it induces transcription of defense genes such as β-1,3-glucanase, chitinase I and other basic-type PR proteins [59]. Real-time PCR showed that EREBP was significantly up-regulated from the early response (2 h) to a highly significant increase at 4, 8 and 12 h with a 11 fold expression (Fig. 2i).

**Cellular stress-related responses**. The multi-component and multi-dimensional mechanisms of plants to recognize and counteract stress conditions result in overlap between abiotic and biotic stress responses [60]. HSP90, stress-responsive cyclophilin, serine hydroxymethyltransferase (SHMT) and other stress-related genes (Table 1) were also up-regulated in tobacco cells following INAP elicitation. SHMT induction has also been observed following elicitation of *Arabidopsis thaliana* by bacterial LPS [13], and is reported to be involved in controlling cell damage caused by abiotic stress as well as HR in plants [61] and to negatively regulate certain SA responsive genes [62]. Interestingly, SHMT has recently been identified as an R gene in soy bean against nematode infection, pointing to a new mechanism of plant resistance to pathogens [63].

The identified Cytochrome P450 showed recognizable high expression from 2 h to 12 h as shown by the qPCR (Fig. 2j). The cytochrome P450 superfamily of enzymes contains heme monooxygenase activity used to oxidize substrates. Cytochromes P450 in plants are involved in a wide range of biosynthetic reactions leading to various fatty acid conjugates, plant hormones and defensive compounds such as phytoalexins [64].

Caffeoyl-CoA O-methyltransferase (CCoAOMT) plays a dominant role in the methylation of the 3-hydroxyl group of caffeoyl CoA, and the CCoAOMT-mediated methylation reaction is essential to channel substrates for 5-methoxylation of hydroxycinnamates for phytoalexin or monolignol synthesis [65]. CCoAOMT might also be involved in the production of a bio-transformation product of INAP, 4’-hexopyranosyloxy-3’-methoxyisonitrosoacetophenone [6].

Flavin monooxygenase (FMO), found to be up-regulated, is an important component of biologically-induced SAR. An Arabidopsis *FMO1* knockout mutant line was fully impaired in the establishment of SAR triggered by avirulent and virulent *Pseudomonas* species [66]. Moreover, at the site of pathogen attack in the presence of the FMO1 gene, an increased level of SA signaling, JA, camalexin and various other defense responses were observed.

**Defense-related proteins**. Activated signal transduction pathways culminate in regulated gene expression, especially by enhancing the expression of genes related to disease resistance, including PR protein encoding genes [67]. INAP induced the expression of various tobacco genes involved in defense responses, such as β-1,3-Glucanase, class II Chitinase, Cysteine proteinase, Thioredoxin, Biotic cell death-associated protein, Defensin, Osmotin, PR-1a, PR1b, etc. (Table 1). During plant defense, β-1,3-Glucanases and Chitinases synergistically hydrolyze β-glucans and chitin, major structural biopolymers that are found in fungal cell walls [68].

qPCR showed that both genes are significantly up-regulated as early responses at the 4 h and 8 h time points post elicitation, before returning to basal levels (Fig. 2k,l). In plants infected with pathogens, β-1,3-Glucanase is part of a long-lasting defense response that is activated during implementation of SAR [29].

Cysteine proteinases, significantly up-regulated at 4 h and 8 h (Fig. 2m), are involved in signaling pathways and in biotic and abiotic stress responses [69]. In animals, and probably in plants, they are involved in the execution of programmed cell death [70].

qPCR showed a significant up-regulated expression of Pheophorbide oxygenase A, an iron-sulfur protein that is encoded by the *accelerated cell death 1* (*ACD1*) gene, from 4 h to 12 h (Fig. 2n). This multi-functional gene product is involved in chlorophyll breakdown [71], but also associated with defense responses against bacteria and limiting spreading necrosis in incompatible interactions (Gene ontology, At3g44880, TAIR). The functionally analogous Biotic cell death-associated protein (a negative regulator of endopeptidase activity, (Uniprot—Gene Ontology; [72]), was also shown to be up-regulated with a slight increase from 2 h to a significant relative expression of 11-fold increase at 8 h (Fig. 2o). It was reported that this protein intervenes at the site of infection and involves the co-ordinated activation of many defense genes that limit the growth of a pathogen in the plant [73].

An important category of genes induced in this study are the Pathogenesis-related proteins 1a (PR-1a) and 1b (PR-1b). PR-1 proteins with antifungal activity are induced during local and systemic resistance, and regarded as markers for SAR [74]. In tobacco cells, PR-1a and PR-1b were found to display differential antifungal activity against *Phytophtora infestans* [75]. In addition, activating the expression of PR-1a and PR-1b by riboflavin was shown to induce protection of tobacco against *Phytophthora parasitica* and *Ralstonia solanacearum* [76]. Here, real time PCR showed that INAP elicitation induced a significant high expression of *PR-1a* and *PR-1b* at 4 h, 12 h and 24 h (Fig. 2p,q) indicating that they were induced exclusively after elicitation, downstream of initial signal transduction events.

Two additional PR proteins identified are Osmotin and Defensin that exhibit antifungal and antibacterial activity [74]. The induction of defense genes encoding β-1,3-Glucanase, Osmotin and PR-1 is controlled by the SA pathway and Chitinase and Defensin by JA/ET-dependent pathways [30]. In addition, PR-1 is generally regarded as a marker for the activation of the SA signaling pathway, whereas Defensin is a marker for JA signaling [77]. Based on the differential expression of these genes, it could be suggested that following INAP elicitation in tobacco, SA, ET and JA-dependent pathways were activated and interacted with each other to induce defense and regulate PR proteins expression.

**Transport, metabolism and energy**. Previous studies on LPS-induced responses in tobacco cells [46,78] found that several metabolism and energy-related proteins were differentially expressed with a shift from normal metabolism to defense metabolism with an increase in demand for energy and biosynthetic capacity provided by primary metabolic pathways [62].

The up-regulated cytochrome c oxidase and NADH-ubiquinone oxidoreductase (Table 1) might contribute to enhanced mitochondrial function and energy production. NADH-ubiquinone oxidoreductase is also a major source of ROS in mitochondria, contributing to cellular redox homeostasis [79].

Activation of defense responses involves changes in the PM permeability that will lead to ion fluxes, requiring carriers /transporters [80]. Various genes involved in transportation were up-regulated during INAP elicitation (Table 1). Of interest is an ATP-binding cassette (ABC) transporter family protein. The functionally diverse ABC transporter family is implicated in ion fluxes, import and export functions and detoxification in support of the interaction of the plant with its environment [81], e.g. the translocation of antimicrobial cargo such as phytoalexins to the extracellular space. INAP-induced changes to secondary metabolism [6] included the up-regulation of genes encoding enzymes involved in synthesis of cinnamates, the building blocks of phytoalexins and lignin precursors.

**Cytoskeleton and cell wall structure**. Signal transduction responses converge at cytoskeleton proteins to contribute to many essential cellular process such as endocytosis, vesicular trafficking and export of newly synthesised defense proteins to extra- and intracellular locations [46]. Tubulin is involved in these cytoskeletal rearrangements and the corresponding gene for alpha-tubulin was found to be up-regulated as listed in Table 1. The cytoskeleton and cell wall-plasma membrane connectivity have been identified as important responsive elements of non-host resistance [82].

Cell wall strengthening is part of the broad spectrum of inducible plant defense responses [83]. Cell wall-related genes induced in this study include putative glycine—and proline-rich proteins, an arabinogalactan-protein, cellulose synthase-like protein H1-like, pectin methylesterase and others as listed in the Table 1. Furthermore, several of these genes listed in the ‘Metabolism’ category (cinnamate-4-hydroxylase, caffeoyl-CoA O-methylesterase and 4-coumarate CoA ligase) are amongst the key enzymes in the synthesis of monolignols, the precursor molecules of polymeric lignin [84]. Based on these findings, even though the changes in their transcript were not confirmed by qPCR, we propose that cell wall-modifying enzymes may form part of the defense-inducing action mechanism of INAP [85].

### INAP-induced responses result in the creation of an enhanced defensive capacity

**Fig 4:**
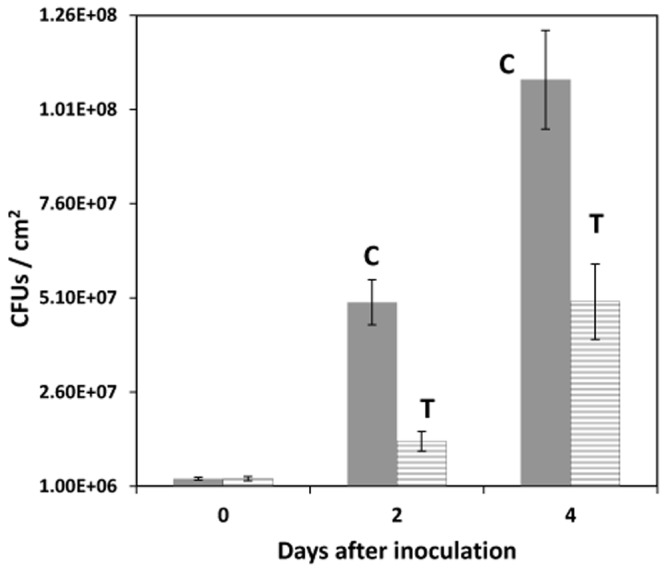
*In planta* growth of *Pseudomonas syringae* pv. *tabaci* in tobacco leaf tissues following pre-conditioning with 1 mM INAP. Graphical representation of bacterial cell counts expressed as colony forming units (CFUs) per square cm obtained from serially diluted extracts after 2 days of growth on King’s B medium. C = Controls, infiltrated with 10 mM MgSO_4_ and T = INAP conditioned tissue, infiltrated with 1 mM INAP dissolved in 10 mM MgSO_4_. Error barrs indicate standard deviation of three biological repeats.

***In planta* growth assays**. We have previously reported that INAP affects the metabolome of treated tobacco cells and found that the observed discriminatory bio-markers included benzoic—or cinnamic acid as well as flavonoid derivatives. INAP thus affects the shikimate–, phenylpropanoid—and flavonoid pathways, the products of which may subsequently contribute to an anti-oxidant and anti-microbial environment *in vivo* [6]. By extending these observations to tobacco leaf tissue, we found that *in planta* growth assays using *Pseudomonas syringae* pv. *tabaci*, indicate that the observed re-programming of the transcriptome in response to INAP treatment resulted in a functional enhanced defensive capacity as reflected by an anti-microbial environment that limited the growth of the pathogen. The results, shown in Fig. 4, illustrates the reduction in cell counts of *Pseudomonas syringae* pv. *tabaci* as determined on day 2 and 4 after inoculation.

## Conclusions

This study has successfully identified INAP-responsive genes known to be involved in several important aspects of plant perception of microbes and induced defense responses to pathogen attack. It was found that qPCR results were in accordance with that of the mRNA differential display. All of the 17 selected genes showed transient, but differential up-regulation between 4 h and 12 h following treatment, decreasing to 24 h post treatment, the last time point of the study. These patterns of expression indicate firstly that INAP can initiate priming / defense response in the plant cells, with the response becoming detectable as early as between 4 h and 8 h, and secondly, that different genes show differential responses to INAP with different induction kinetic profiles. Here, it needs to be taken into account that primed plants generally do not activate defenses directly, and that the primed condition is based on accumulation of signal molecules, post-translational modifications and proteins that remain inactive until re-encountering pathogens able to cause disease [86]. The re-programming of the transcriptome to create an anti-microbial environment was effective in limiting the growth of the pathogen *Pseudomonas syringae* pv. *tabaci*.

It can be concluded that INAP is able to induce several genes of importance to signaling, priming and defense-related responses in plants (summarized in Fig. 3). INAP is a novel chemical activator of plant defenses and its mode of action was previously unknown. Through the identification of the differentially expressed genes, the mode of action of INAP is now better understood, allowing further investigations into its possible use in novel / alternative crop protection strategies.

## Supporting Information

S1 Fig Representative agarose gel electrophoresis of PCR products obtained through ACP-DDRT-PCR amplification (A) and re-amplification of excised bands (B). (DOCX)

S1 Table Primer sequences used in ACP-DDRT-PCR. (DOC)

S2 Table Primer sequences for qRT-PCR analysis of expression kinetics of selected INAP-responsive genes. (DOC)

S3 Table Accession numbers of selected candidate genes differentially expressed in tobacco cell suspensions in response to INAP treatment. (DOCX)

S4 Table Summary of other genes obtained from the contig and singleton data identified by both BLAST-N and BLAST-X. (DOC)
