# Supplementary material for: Isonitrosoacetophenone Drives Transcriptional Reprogramming in Nicotiana tabacum Cells in Support of Innate Immunity and Defense
Source: PLoS One. 2015 Feb 6;10(2):e0117377. doi: 10.1371/journal.pone.0117377 (PMC4319752; doi:10.1371/journal.pone.0117377)

**Isonitrosoacetophenone** **Drives Transcriptional Reprogramming in *Nicotiana tabacum* Cells in Support of Innate Immunity and Defense**

**Arnaud T Djami-Tchatchou^1¶^, Mmapula Peggy Maake^1¶^, Lizelle A Piater and Ian A Dubery***

**Figure S1. Representative agarose gel electrophoresis of PCR products obtained through ACP-DDRT-PCR amplification.**

**A**: ACP-DDRT-PCR products were obtained using 1-60 ACP arbitrary primer pairs. Bands represent amplicons from genes differentially expressed at the time points investigated. The number of amplicons is dependant on the primer sets used (Table S1) and the different patterns obtained can be ascribed to different expression kinetics exhibited by the corresponding genes.

**B**: Size range (250-350 bp) of re-amplification products of differentially expressed amplicons obtained from the cDNA of up-regulated genes excised from the gel and re-amplified with universal forward and reverse primers with sequences complementary to the 5´ end of the ACP-primers used in the ACP-PCR (Table S1). M represents molecular DNA molecular size ladders (75-700 bp).


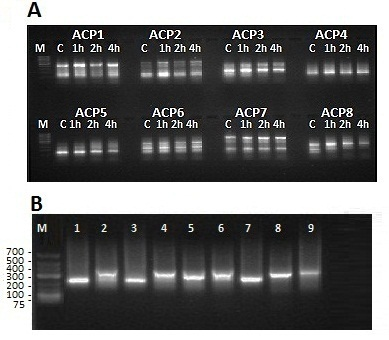

Supplement: S1 Fig — (DOCX) [file pone.0117377.s001.docx]
