# Supplementary material for: Isonitrosoacetophenone Drives Transcriptional Reprogramming in Nicotiana tabacum Cells in Support of Innate Immunity and Defense
Source: PLoS One. 2015 Feb 6;10(2):e0117377. doi: 10.1371/journal.pone.0117377 (PMC4319752; doi:10.1371/journal.pone.0117377)
Supplement: S1 Table — (DOC) [file pone.0117377.s002.doc]

**Table S1: Primer sequences used in ACP-DDRT-PCR**

**Anchored ACP Oligo dT Primers**

For RT: dT-ACP1: 5′-CTGTGAATGCTGCGACTACGATXXXXX(T)18 -3′

For PCR: dT-ACP2: 5′-CTGTGAATGCTGCGACTACGATXXXXX(T)15 -3′

**Arbitrary Primers**

ACP1 : 5′-GTCTACCAGGCATTCGCTTCATXXXXXGCCATCGACC-3′

ACP2 : 5′-GTCTACCAGGCATTCGCTTCATXXXXXAGGCGATGCC-3′

ACP3 : 5′-GTCTACCAGGCATTCGCTTCATXXXXXCCGGAGGATG-3′

ACP4 : 5′-GTCTACCAGGCATTCGCTTCATXXXXXGCTGCTCGCG-3′

ACP5 : 5′-GTCTACCAGGCATTCGCTTCATXXXXXAGTGCGCTCG-3′

ACP6 : 5′-GTCTACCAGGCATTCGCTTCATXXXXXGGCCACATCG-3′

ACP7 : 5′-GTCTACCAGGCATTCGCTTCATXXXXXCTGCGGATCG-3′

ACP8 : 5′-GTCTACCAGGCATTCGCTTCATXXXXXGGTCACGGAG-3′

ACP9 : 5′-GTCTACCAGGCATTCGCTTCATXXXXXGATGCCGCTG-3′

ACP10: 5′-GTCTACCAGGCATTCGCTTCATXXXXXTGGTCGTGCC-3′

ACP11: 5′-GTCTACCAGGCATTCGCTTCATXXXXXCTGCAGGACC-3′

ACP12: 5′-GTCTACCAGGCATTCGCTTCATXXXXXACCGTGGACG-3′

ACP13: 5′-GTCTACCAGGCATTCGCTTCATXXXXXGCTTCACCGC-3′

ACP14: 5′-GTCTACCAGGCATTCGCTTCATXXXXXGCAAGTCGGC-3′

ACP15: 5′-GTCTACCAGGCATTCGCTTCATXXXXXCCACCGTGTG-3′

ACP16: 5′-GTCTACCAGGCATTCGCTTCATXXXXXGTCGACGGTG-3′

ACP17: 5′-GTCTACCAGGCATTCGCTTCATXXXXXCAAGCCCACG-3′

ACP18: 5′-GTCTACCAGGCATTCGCTTCATXXXXXCGGAGCATCC-3′

ACP19: 5′-GTCTACCAGGCATTCGCTTCATXXXXXCTCTGCGAGC-3′

ACP20: 5′-GTCTACCAGGCATTCGCTTCATXXXXXGACGTTGGCG-3′

ACP21-40: Propriety information : SeeGene, Inc., Korea.

Universal upstream primer: 5´-GTCTACCAGGCATTCGCTTCAT-3´

Universal downstream primer: 5´-CTGTGAATGCTGCGACTCGAT-3´.
