# Supplementary material for: Isonitrosoacetophenone Drives Transcriptional Reprogramming in Nicotiana tabacum Cells in Support of Innate Immunity and Defense
Source: PLoS One. 2015 Feb 6;10(2):e0117377. doi: 10.1371/journal.pone.0117377 (PMC4319752; doi:10.1371/journal.pone.0117377)
Supplement: S2 Table — (DOC) [file pone.0117377.s003.doc]

**Table S2**. Primer sequences for qRT-PCR analysis of expression kinetics of selected INAP-responsive genes.

| **Putative identity/sequence source** | **Product**  **length** | **Primer Sequence**  **(5’-3’)** | | **Tm (^o^C)** |
| --- | --- | --- | --- | --- |
| 18S ribosomal RNA gene (AY079155.1) | 150 | Forward Reverse | GGCAAATAGGAGCCAATGAA  GGGGTGAACCAAAAGCTGTA | 57.0 |
| Elongation factor α gb| D63396.1| | 107 | Forward Reverse | ACC AGA TCA ATG AGC CCA AG  AAG AGC TTC GTG GTG CAT CT | 60.0 |
| β-1,3-glucanase (HSZW1U101BMRIS) | 184 | Forward Reverse | GCTTCTCTCGTAAGTGAGATGTGAGC  ACTCAGAGTTGCATCGTCACTGGA | 58.5 59.7 |
| Pre-pro-cysteine proteinase [(contig00026)](http://www.ncbi.nlm.nih.gov/nucleotide/19850?report=genbank&log$=nucltop&blast_rank=1&RID=4V88EKR601N) | 80 | Forward Reverse | TCTACTGTGACTGCTGCCCATACT  CCCTTGATCACAGACAACATTCCTGG | 59.2  59.2 |
| Cyclophilin (Contig00001) | 80 | Forward Reverse | TAGCAGAGTTGACCACAGTCAGCA  AAGAAGGCTGAGGCTGTTGGATCT | 59.7 60.0 |
| EREBP (Contig00040) | 102 | Forward Reverse | AGCATTTCCACCATCCTGTGTTGC  TCCCAGATGAAGTTCTTGCAGATCCC | 60.0 60.1 |
| Thioredoxin (Contig00045) | 110 | Forward Reverse | GCATCCATATGAGGTGATGACTGC  GACCAAACTCAGCCGGTACATTAG | 57.5 57.2 |
| HSP90 [(Contig00048)](http://www.ncbi.nlm.nih.gov/nucleotide/392465168?report=genbank&log$=nucltop&blast_rank=2&RID=4V8C796R01N) | 105 | Forward Reverse | TCCCTTCCTCGTCTTTCTTCTCCT  ACTCCGAGTTCATCAGCTACCCAA | 58.8 59.4 |
| SAR1(GTPases) (Contig00050) | 150 | Forward Reverse | ACCTAGACAAGAGAAACTATAGCCC  TGGAGAAGGCTTCAGATGGATGTC | 55.5  58.2 |
| Chitinase, stress-related gi|62719020:1-272 | 85 | Forward Reverse | TCACAACTTCAACTACGGACAGCC  TTTCTCCTCTTCGTAACCGTACCC | 58.8 57.8 |
| ACRE 261 (Contig00093) | 119 | Forward Reverse | ACAGTTCAACGATGGAGGGACCAT  TAACCTGAGCTTGCATTGCATCCC | 59.9 59.6 |
| Biotic cell death-associated protein (BIOTHSZW1U101A3L23) | 170 | Forward Reverse | TCGAAGGGTGCTTAGAAGATGGCA  TTGGACGATTTACAGCCAAACGCC | 60.3 59.9 |
| Pheophorbide oxygenase A (HSZW1U101A9XP5) | 82 | Forward Reverse | GAGAAGCTGCGTGTTATCTTGGCT  AATTCTTCTGCAGTTCGTGGAGGG | 59.1 59.0 |
| Cytochrome P450 (HSZW1U101BQZCB) | 84 | Forward Reverse | AGGACCACTACTAGTCCCACACGA  ACAAGTAACATGGTTCCTCCGGGT | 60.4 59.9 |
| NPR1 gb|AF480488.1| | 94 | Forward Reverse | CTCAAGTTGATGGCACTTCT  GGAGCCTCGTTCAAATCTAC | 60.0 |
| RAR1 gb|AF480487.1| | 92 | Forward Reverse | GTTCACAACCCAGAGAAGTAA  AGGATGGCATTGCTGTATATC | 60.0 |
| SGT1 gb|AF516180.1| | 125 | Forward Reverse | GAAGCTGGAGAACTACCTAATC  AGGTTGACAGTTGGTTGAG | 60.0 |
| PR1a [gb|JN247448.1](http://www.ncbi.nlm.nih.gov/nucleotide/343174747?report=genbank&log$=nuclalign&blast_rank=1&RID=D260AHTT014)| | 98 | Forward Reverse | ACAAGACTATTTGGATGCCC  GTATGGACTTTCGCCTCTAT | 60.0 |
| PR-1b gb|X66942.1| | 101 | Forward Reverse | ATGGGATACTCCACAACAT  TTACTAGACATCAGTTGGAAGT | 60.0 |
