# Supplementary material for: Isonitrosoacetophenone Drives Transcriptional Reprogramming in Nicotiana tabacum Cells in Support of Innate Immunity and Defense
Source: PLoS One. 2015 Feb 6;10(2):e0117377. doi: 10.1371/journal.pone.0117377 (PMC4319752; doi:10.1371/journal.pone.0117377)
Supplement: S3 Table — (DOCX) [file pone.0117377.s004.docx]

**Table S3.** Accession numbers of selected candidate genes differentially expressed in tobacco cell suspensions in response to INAP treatment.

| **Name** | **Accession number of similar sequence** | **Similar sequence from database BLASTN** | **E-value** | **Max identity %** |
| --- | --- | --- | --- | --- |
| **SIGNAL PERCEPTION AND TRANSDUCTION** | | | | |
| HSZW1U101AA3F1 | JF919621.1 | *Nicotiana attenuata* lectin-domain receptor-like kinase (lecRK1) mRNA, lecRK1.1 allele, complete cds | 7.00E-16 | 78 |
| HSZW1U101BL2SE | ACN59240.1 | *Arabidopsis thaliana* leucine-rich repeat receptor-like protein kinase | 5.00E-04 | 92 |
| HSZW1U101BRXWL | EU450800.1 | *Glycine max* NB-LRR type disease resistance protein Rps1-k-1 (Rps1-k-1) genes, complete cds | 0.006 | 80 |
| HSZW1U101A7DWC | NM_101103.3 | *Arabidopsis thaliana* putative calcium-binding protein CML13 (AT1G12310) mRNA | 9.00E-19 | 75 |
| HSZW1U101BQVWC | X58578.1 | *Pinus sylvestris* mRNA for CuZn superoxide dismutase, clone PS3 | 7.00E-05 | 85 |
| HSZW1U101BVY32 | AB052964.1 | *Nicotiana tabacum* MAPK gene for wound induced protein kinase (WIPK), complete cds | 2.00E-12 | 80 |
| HSZW1U101BZHJA | Y11212.1 | *Nicotiana tabacum* mRNA for 14-3-3-like protein | 2.00E-08 | 85 |
| ZW1U101BX9I3 | DQ359730.1 | *Nicotiana attenuata* JAR1-like protein (JAR6) mRNA, complete cds | 0.084 | 93 |
| HSZW1U101BN2YF | AJ440219.1 | *Oryza sativa* a8 gene for plasma membrane H+-ATPase | 0.33 | 100 |
| Contig 00023 | NM_001247571.1 | *Solanum lycopersicum* protein phosphatase 2C (DIG3), mRNA | 9.00E-38 | 91 |
| Contig00050 | gi\|1340114\| / X97967.1 | *Nicotiana tabacum* mRNA for GTP-binding protein, GTPase SAR1 | 4.00E-123 | 100 |
| Contig00067 | [gi\|224815411\|FJ755907.1](http://www.ncbi.nlm.nih.gov/nucleotide/224815411?report=genbank&log$=nucltop&blast_rank=2&RID=4VBSMMZD01N) | *Nicotiana benthamiana* Rab GDP dissociation inhibitor (GDI) mRNA, complete cds | 2.00E-101 | 98 |
| UCTSEG001 | AF012823 | *Nicotiana tabacum* Rho GDP dissociation inhibitor (GDI) mRNA, complete cds | 6.00E-145 | 91 |
| **REGULATION** | | | | |
| HSZW1U101BSD87 | gb\|AF516180.1\| | *Nicotiana attenuata* SGT1 mRNA, complete cds | 2.00E-09 | 98 |
| HSZW1U101BUK1L | AB644218.1 | *Nicotiana tabacum* NtARL8b mRNA for ADP-ribosylation factor-like 8b, complete cds | 8.00E-42 | 100 |
| HSZW1U101A20SL | AF390556.1 | *Nicotiana tabacum* Bax inhibitor 1 (BI-1) mRNA, complete cds | 8.00E-87 | 99 |
| HSZW1U101A79RR | AB610787.1 | *Petunia x hybrida* PhRR1 mRNA for type-A response regulator, complete cds | 1.00E-22 | 87 |
| HSZW1U101BPEIK | gb\|AF480488.1\| | *Populus trichocarpa* NPR1/NIM1-like regulatory protein, mRNA | 0.18 | 84 |
| HSZW1U101BW4OV | NM_001247550.1 | *Solanum lycopersicum* ripening regulated protein DDTFR19, mRNA | 5.00E-13 | 91 |
| **TRANSCRIPTION** | | | | |
| HSZW1U101BWSE1 | AF153278.1 | *Nicotiana tabacum* DNA-directed RNA polymerase IIb (NT193) mRNA, complete cds | 5.00E-110 | 96 |
| HSZW1U101A889V | D86722.1 | *Nicotiana tabacum* mRNA for TATA binding protein (TBP), complete cds | 1.00E-90 | 98 |
| HSZW1U101A5J9U | AF262733.2 | *Arabidopsis thaliana* putative transcription factor MYB108 mRNA, complete cds | 5.00E-06 | 92 |
| Contig 00113 | AAC24369.1 | *Arabidopsis thaliana* bZIP-like transcription factor protein | 0.002 | 52 |
| Contig00040 | gi\|89258367 | *Capsicum annuum* putative ethylene-responsive element binding protein (JERF1) mRNA, complete cds | 6.00E-96 | 87 |
| ZW1U101BMIKZ | ADL36790.1 | *Malus x domestica* NAC domain class transcription factor | 0.002 | 71 |
| HSZW1U101A6PQ8 | AB041520.1 | *Nicotiana tabacum* mRNA for WRKY transcription factor NtEIG-D48, complete cds | 2.00E-07 | 97 |
| HSZW1U101BOPYS | AJ937310.1 | *Juglans regia* mRNA for putative Cys2-His2 zinc finger transcription factor (zfp2 gene) | 2.00E-08 | 76 |
| HSZW1U101BRXU6 | FJ809943.1 | *Vitis vinifera* transcription factor APETALA2 (AP2) mRNA, complete cds | 9.00E-05 | 85 |
| **PROTEIN UBIQUITINATION** | | | | |
| Contig00154 | gi\|225432555\|XM_002280892.1 | *Vitis vinifera* NEDD8-activating enzyme E1 catalytic subunit-like (LOC100256207), mRNA | 4.00E-18 | 87 |
| Contig 00107 | gi\|388281865\| / AB721415. | *Pyrus pyrifolia* var*. culta* mRNA for putative E3 ubiquitin ligase, partial cds | 3.00E-09 | 100 |
| HSZW1U101A1UQD | AY486137.1 | *Capsicum annuum* ubiquitin-conjugating protein mRNA, complete cds | 2.00E-12 | 77 |
| Contig 00052 | gi\|321149956\| / GU594243.1 | *Solanum nigrum* clone 110 ubiquitin extension protein (Ubi2) mRNA, complete cds | 1.00E-16 | 96 |
| Contig 00102 | gi\|255565345 / XM_002523618.1 | *Ricinus communis* 26S protease regulatory subunit 6b, putative, mRNA | 5.00E-12 | 96 |
| Contig00101 | gi\|255547771\|XM_002514897.1 | *Ricinus communis* proteasome subunit beta type 5,8, putative, mRNA | 4.00E-20 | 82 |
| **PROTEIN SYNTHESIS AND FOLDING** | | | | |
| Contig 00060 | gi\|2708531\| / AF029351.1 | *Nicotiana tabacum* putative RNA binding protein (QRRBP-1) mRNA, partial cds | 3.00E-105 | 93 |
| Contig00019 | gi\|7673358\|AF190657.1 | *Nicotiana tabacum* clone 7 poly(A)-binding protein (PABP) mRNA, partial cds | 3.00E-151 | 98 |
| Contig 00020 | gi\|1009231\| / L38828.1 | *Nicotiana tabacum* EF-1-alpha-related GTP-binding protein (SUP1) mRNA, complete cds | 4.00E-144 | 96 |
| Contig 00001 | gi\|46404795\| / AY368274.1 | *Nicotiana tabacum* cyclophilin-like (CYP1) mRNA, complete sequence | 0 | 97 |
| **VESICLES AND TRANSPORTATION** | | | | |
| HSZW1U101BY2Q8 | XR_137452.1 | *Glycine max* ABC transporter B family member 25-like (LOC100810510), mRNA | 2.00E-15 | 85 |
| Contig00034 | gi\|126363775\|AB286962.1 | *Nicotiana tabacum* NtMATE2 mRNA for multi antimicrobial extrusion family protein, complete cds | 1.00E-149 | 98 |
| Contig 00055 | gi\|82623434\| / DQ241861.1 | *Solanum tuberosum* clone 021G11 translocon-associated protein (TRAP) beta family protein, mRNA, complete cds | 2.00E-81 | 93 |
| HSZW1U101A0XGV | AB721408.1 | *Pyrus pyrifolia* var*. culta*  mRNA for putative tonoplast intrinsic protein (TIP) 1, partial cds | 7.00E-04 | 100 |
| Contig 00191 | NP_182045.1 | *Arabidopsis thaliana* Golgi SNARE 12 protein | 4.00E-04 | 34 |
| HSZW1U101A38AJ | XM_002264608.2 | *Vitis vinifera* endoplasmic reticulum-Golgi intermediate compartment protein 3-like (LOC100267365), mRNA | 9.00E-04 | 83 |
| HSZW1U101BXHUQ | AT1G36050, [NM_103301.6](http://www.ncbi.nlm.nih.gov/nucleotide/240254209?report=genbank&log$=nucltop&blast_rank=2&RID=4Y9ME1UR01N) | *Arabidopsis thaliana* endoplasmic reticulum vesicle transporter protein | 5.00E-10 | 86 |
| HSZW1U101BTP0Z | Z71395.1 | *Nicotiana plumbaginifolia* mRNA for calreticulin | 9.00E-19 | 95 |
| **STRESS-RELATED RESPONSE** | | | | |
| Contig 00048 | AB689674.1 | *Nicotiana tabacum* NtHsp90 mRNA for heat shock protein 90, complete cds | 4.00E-91 | 90 |
| Contig 00118 | AJ937852.1 | *Nicotiana tabacum* partial mRNA for putative glutathione S transferase (GST1 gene), clone EBR-52 | 1.00E-57 | 87 |
| Contig00147 | gi\|218293\|D10524.1 | *Nicotiana tabacum* mRNA for glutathione S-transferase, complete cds | 2.00E-53 | 98 |
| HSZW1U101BSQQM | NM_115259.4 | *Arabidopsis thaliana* universal stress protein (USP) family protein (AT3G53990) mRNA, complete cds | 5.00E-31 | 79 |
| HSZW1U101A8TC9 | GU994207.1 | *Nicotiana tabacum* cytosolic class I small heat shock protein 3A (sHSP3A) gene, promoter region and complete cds | 9.00E-98 | 99 |
| Contig00045 | gi\|21912926\|AJ309009.2 | *Nicotiana tabacum* mRNA for thioredoxin peroxidase | 1.00E-104 | 100 |
| HSZW1U101A4ONF | XM_002285569.2 | *Vitis vinifera* serine hydroxymethyltransferase, mitochondrial-like (LOC100245411), mRNA | 7.00E-15 | 87 |
| HSZW1U101A911V | U92087.1 | *Solanum commersonii* stress responsive cyclophilin (SCCYP1) mRNA, complete cds | 3.00E-05 | 97 |
| **DEFENSE RESPONSE** | | | | |
| Contig00093 | gi\|56544481 | *Nicotiana tabacum* Avr9/Cf-9 rapidly elicited protein 261 (ACRE261) mRNA, partial cds | 3.00E-40 | 83 |
| HSZW1U101A3L23 | AF208022.1 | *Nicotiana glutinosa* biotic cell death-associated protein (CDM1) mRNA, complete | 8.00E-37 | 75 |
| HSZW1U101BMRIS | S44870.1 | *Nicotiana tabacum* basic β-1,3-glucanase {clone FB7-5(1)} | 1.00E-15 | 96 |
| HSZW1U101BVGTM | gi\|62719020:1-272 | *Elaeis guineensis* class II chitinase (CHI2) mRNA, partial cds | 3.00E-06 | 92 |
| Contig00056 | gi\|62719020\|AJ880385.1 | *Nicotiana tabacum* partial mRNA for putative stress related chitinase (cht STR1 gene), clone CHO3E10 | 1.00E-117 | 98 |
| Contig00026 | Z13964.1 | *Nicotiana tabacum* mRNA for pre-pro-cysteine proteinase | 8.00E-159 | 98 |
| Contig 00183 | NP_001031236.1 | *Arabidopsis thaliana* defensin-like protein 226 | 2.00E-04 | 44 |
| HSZW1U101A9XP5 | ABY19384.1 | *Nicotiana tabacum* pheophorbide A oxygenase 1 mRNA, complete | 4.00E-109 | 99 |
| HSZW1U101BZ7E8 | S44889.1 | *Nicotiana tabacum* osmotin pathogenesis-related protein homolog | 4.00E-07 | 100 |
| HSZW1U101BMIXP | AB625594.1 | *Nicotiana tabacum* NtLTP3 gene for lipid transfer protein, complete cds | 2.00E-41 | 93 |
| **METABOLISM AND ENERGY** | | | | |
| HSZW1U101BQZCB | AB006654.1 | *Nicotiana tabacum* TCP1 mRNA for cytochrome P450, complete cds | 4.00E-94 | 99 |
| Contig 00104 | gi\|212960468\| / FJ410448.1 | *Betula luminifera* 4-coumarate coenzyme A ligase (4CL) mRNA, complete cds | 0.003 | 89 |
| Contig 00024 | gi\|388281857\| / AB721411.1 | *Pyrus pyrifolia* var. *culta* mRNA for putative caffeoyl-CoA O-methyltransferase, partial cds | 1.00E-05 | 92 |
| HSZW1U101BLI2Y | EU123531.1 | *Rubus coreanus* cinnamate-4-hydroxylase mRNA, complete cds | 8.00E-08 | 87 |
| HSZW1U101BYF4L | AY039108.1 | *Petunia x hybrid* flavin monoxygenase (FMO)-like protein mRNA, fzy-R27 allele, complete cds | 0.081 | 81 |
| HSZW1U101A5TAG | X83229.1 | *Nicotiana tabacum* mRNA for 1-aminocyclopropane-1-carboxylate oxidase | 3.00E-47 | 81 |
| HSZW1U101A2D7J | AB555734.1 | *Gardenia jasminoides* GjUGT4 mRNA for UDP-glucose glucosyltransferase, complete cds | 7.00E-04 | 100 |
| HSZW1U101BUD3N | AB072919.1 | *Nicotiana tabacum* NtGT2 mRNA for glucosyltransferase, complete cds | 2.00E-51 | 94 |
| HSZW1U101A18CH | XM_003588363.1 | *Medicago truncatula* cytochrome c oxidase subunit (MTR_1g006950) | 1.00E-16 | 98 |
| HSZW1U101A0KHO | X96671.1 | *Solanum tuberosum* mRNA for NADH-ubiquinone oxidoreductase subunit | 1.00E-23 | 78 |
| **CELL WALL-RELATED AND CYTOSKELETON** | | | | |
| Contig00032 | gi\|19742\|X60007.1 | *Nicotiana sylvestris* mRNA for glycine rich protein 2 (GRP2) | 5.00E-161 | 98 |
| Contig 00006 | gi\|10798751\|AB041516.1 | *Nicotiana tabacum* mRNA for proline-rich protein EIG-I30, complete cds | 1.00E-178 | 100 |
| Contig 00015 | XP_003526337.1 | *Glycine max* cellulose synthase-like protein H1-like | 1.00E-05 | 76 |
| HSZW1U101A409Z | AJ401158.1 | *Nicotiana tabacum* partial mRNA for pectin methylesterase | 3.00E-34 | 81 |
| HSZW1U101BPTXB | AJ853473.1 | *Nicotiana glauca* mRNA for putative arabinogalactan-protein precursor (arg gene) | 3.00E-47 | 82 |
| Contig00100 | gi\|388281855\|AB721410.1 | *Pyrus pyrifolia* var. *culta* mRNA for putative annexin, partial cds | 2.00E-04 | 94 |
| Contig00144 | gi\|505143\|D13951.1 | *Nicotiana tabacum* gene for extensin, complete cds | 2.00E-73 | 100 |
| Contig 00173 | gi\|17402468\| / AJ421412.1 | *Nicotiana tabacum* mRNA for alpha-tubulin (tubA2 gene) | 4.00E-36 | 100 |
