# Supplementary material for: Isonitrosoacetophenone Drives Transcriptional Reprogramming in Nicotiana tabacum Cells in Support of Innate Immunity and Defense
Source: PLoS One. 2015 Feb 6;10(2):e0117377. doi: 10.1371/journal.pone.0117377 (PMC4319752; doi:10.1371/journal.pone.0117377)
Supplement: S4 Table — (DOC) [file pone.0117377.s005.doc]

**Table S4.** Summary of other genes obtained from the contig and singleton data identified by both BLAST-N and BLAST-X.

| **Name** | | **BLAST-N**  (Accession number of similar sequence) | **E-value** | **Max Identity**  % | | | **BLAST-X**  (Accession number of similar sequence) | | **E-value** | **Max Identity**  % |
| --- | --- | --- | --- | --- | --- | --- | --- | --- | --- | --- |
| **SIGNAL TRANSDUCTION** | | | | | | | | | | |
| Contig00061 | | *Arabidopsis lyrata* subsp*. lyrata* kinase family protein, Mrna. [gi|297829911|XM_002882792.1](http://www.ncbi.nlm.nih.gov/nucleotide/297829911?report=genbank&log$=nucltop&blast_rank=4&RID=4V8FEUY701N) | 2.00E-26 | 84 | | | Casein kinase, putative [*Ricinus communis*] [XP_002531638.1](http://www.ncbi.nlm.nih.gov/protein/255581670?report=genbank&log$=prottop&blast_rank=3&RID=4VPGPFE601N) | | 6.00E-20 | 95 |
| Contig00040 | | *Capsicum annuum* putative ethylene-responsive element binding protein (JERF1) mRNA, complete cds [gi|89258367|DQ412079.1](http://www.ncbi.nlm.nih.gov/nucleotide/89258367?report=genbank&log$=nucltop&blast_rank=1&RID=4V8AJSEW01N) | 6.00E-96 | 87 | | | Ethylene-responsive-element-binding factor 3 [*Petunia x hybrida*] [ADP37418.1](http://www.ncbi.nlm.nih.gov/protein/310892552?report=genbank&log$=prottop&blast_rank=1&RID=4VKUYFRB01N) | | 1.00E-30 | 91 |
| Contig00067 | | *Nicotiana benthamiana* Rab GDP dissociation inhibitor (GDI) mRNA, complete cds [gi|224815411|FJ755907.1](http://www.ncbi.nlm.nih.gov/nucleotide/224815411?report=genbank&log$=nucltop&blast_rank=2&RID=4VBSMMZD01N) | 2.00E-101 | 98 | | | Rab GDP dissociation inhibitor [*Nicotiana benthamiana*]*.* [ACN65853.1](http://www.ncbi.nlm.nih.gov/protein/224815412?report=genbank&log$=prottop&blast_rank=1&RID=4VPGPFE601N) | | 9.00E-31 | 100 |
| Contig00166 | | *Nicotiana tabacum* small Ras-like GTP-binding protein (Ran-A2) mRNA, 3' end [gi|496269|L16786.1](http://www.ncbi.nlm.nih.gov/nucleotide/496269?report=genbank&log$=nucltop&blast_rank=2&RID=4VBY80DF01N) | 5.00E-42 | 91 | | | Hypothetical protein MTR_7g013400 [*Medicago truncatula*] [XP_003621443.1](http://www.ncbi.nlm.nih.gov/protein/357502309?report=genbank&log$=prottop&blast_rank=1&RID=4VSA1YXT016) | | 7.00E-05 | 83 |
| Contig00023 | | *Solanum lycopersicum* protein phosphatase 2C (DIG3), mRNA. [gi|350536386|NM_001247571.1](http://www.ncbi.nlm.nih.gov/nucleotide/350536386?report=genbank&log$=nucltop&blast_rank=4&RID=4V88EKR601N) | 9.00E-38 | 91 | | | Protein phosphatase 2C [*Solanum lycopersicum*] [NP_001234500.1](http://www.ncbi.nlm.nih.gov/protein/350536387?report=genbank&log$=prottop&blast_rank=1&RID=4VKTA5WY01N) | | 2.00E-19 | 86 |
| HSZW1U101A7DWC | | *Arabidopsis thaliana* putative calcium-binding protein CML13 (AT1G12310) mRNA, [NM_101103.3](http://www.ncbi.nlm.nih.gov/nucleotide/42561944?report=genbank&log$=nucltop&blast_rank=22&RID=4YH0YMDG01N) | 9.00E-19 | 75 | | | Putative calcium-binding protein CML14 [*Arabidopsis thaliana*] [NP_176470.1](http://www.ncbi.nlm.nih.gov/protein/15221593?report=genbank&log$=prottop&blast_rank=6&RID=50HFA6C801N) | | 1.00E-14 | 84 |
| HSZW1U101BNWXO | | *Medicago sativa* mitogen-activated protein kinase (MAPK) mRNA, complete cds [DQ417654.1](http://www.ncbi.nlm.nih.gov/nucleotide/89520688?report=genbank&log$=nucltop&blast_rank=2&RID=4XES38XB016) | 0.074 | 93 | | | PREDICTED: serine/threonine-protein kinase AFC3-like [*Glycine max*] [XP_003526469.1](http://www.ncbi.nlm.nih.gov/protein/356515564?report=genbank&log$=prottop&blast_rank=1&RID=50J5YSW301N) | | 8.00E-04 | 36 |
| UCTSEG001 | | *Nicotiana tabacum* RhoGDP dissociation inhibitor (GDI) mRNA, complete cds AF012823 | 6.00E-145 | 91 | | | RhoGDP dissociation inhibitor (GDI) [*Nicotiana tabacum*] AAB80717.1 | | 900E-13 | 100 |
| HSZW1U101BN2YF | | *Oryza sativa* a8 gene for plasma membrane H+-ATPase [AJ440219.1](http://www.ncbi.nlm.nih.gov/nucleotide/20302444?report=genbank&log$=nucltop&blast_rank=29&RID=4YFW5Z5E014) | 0.33 | 100 | | | NADH dehydrogenase subunit F [*Juncus decipiens f. spiralis*] [AAQ12057.1](http://www.ncbi.nlm.nih.gov/protein/33333917?report=genbank&log$=prottop&blast_rank=3&RID=50K7EHT5016) | | 0.046 | 41 |
| HSZW1U101A0WUX | | *Pyrus pyrifolia* var*. culta* mRNA for putative auxin-responsive family protein, partial cds [AB721413.1](http://www.ncbi.nlm.nih.gov/nucleotide/388281861?report=genbank&log$=nucltop&blast_rank=1&RID=4X2F7FCN014) | 7.00E-05 | 100 | | | Putative auxin-repressed protein, partial [*Pyrus pyrifolia* var. *culta*] [BAM15888.1](http://www.ncbi.nlm.nih.gov/protein/388281860?report=genbank&log$=prottop&blast_rank=1&RID=4Z60DZKK016) | | 0.003 | 91 |
| **PROTEIN DEGRADATION AND UBIQUITINATION** | | | | | | | | | | |
| Contig00107 | *Pyrus pyrifolia* var*. culta* mRNA for putative E3 ubiquitin ligase, partial cds [gi|388281865|AB721415.1](http://www.ncbi.nlm.nih.gov/nucleotide/388281865?report=genbank&log$=nucltop&blast_rank=2&RID=4VBU3ETS01N) | | 3.00E-09 | | | 100 | | Putative E3 ubiquitin ligase, partial [*Pyrus pyrifolia* var*. culta*] [BAM15891.1](http://www.ncbi.nlm.nih.gov/protein/388281866?report=genbank&log$=prottop&blast_rank=1&RID=4VPNUXBT014) | 2.00E-04 | 68 |
| Contig00101 | *Ricinus communis* proteasome subunit beta type 5,8, putative, mRNA. [gi|255547771|XM_002514897.1](http://www.ncbi.nlm.nih.gov/nucleotide/255547771?report=genbank&log$=nucltop&blast_rank=4&RID=4VBU3ETS01N) | | 4.00E-2 | | | 82 | | 20S proteasome beta 5 subunit [*Triticum aestivum*] [AAU82107.1](http://www.ncbi.nlm.nih.gov/protein/52548240?report=genbank&log$=prottop&blast_rank=2&RID=4VPJUZEH014) | 6.00E-15 | 79 |
| Contig00102 | *Ricinus communis* 26S protease regulatory subunit 6b, putative, mRNA [gi|255565345|XM_002523618.1](http://www.ncbi.nlm.nih.gov/nucleotide/255565345?report=genbank&log$=nucltop&blast_rank=4&RID=4VBU3ETS01N) | | 5.00E-12 | | | 96 | | Putative 26S proteasome regulatory complex protein [*Sandersonia aurantiaca*] [ACA58350.1](http://www.ncbi.nlm.nih.gov/protein/169635147?report=genbank&log$=prottop&blast_rank=1&RID=4VPJUZEH014) | 2.00E-06 | 93 |
| Contig00052 | *Solanum nigrum* clone 110 ubiquitin extension protein (Ubi2) mRNA, complete cds [gi|321149956|GU594243.1](http://www.ncbi.nlm.nih.gov/nucleotide/321149956?report=genbank&log$=nucltop&blast_rank=5&RID=4V8C796R01N) | | 1.00E-16 | | | 96 | | Ubiquitin fusion protein [*Oryza sativa Indica Group*][ABR25730.1](http://www.ncbi.nlm.nih.gov/protein/149391425?report=genbank&log$=prottop&blast_rank=3&RID=4VKWP2XC01N) | 6.00E-04 | 100 |
| Contig00072 | PREDICTED: *Vitis vinifera* proteasome subunit beta type-5-like (LOC100261458), mRNA [gi|359473723|XM_002264792.2](http://www.ncbi.nlm.nih.gov/nucleotide/359473723?report=genbank&log$=nucltop&blast_rank=4&RID=4VBSMMZD01N) | | 5.00E-20 | | | 82 | | 20S proteasome beta subunit 5 [*Citrus maxima*] [ACP43316.1](http://www.ncbi.nlm.nih.gov/protein/227937351?report=genbank&log$=prottop&blast_rank=1&RID=4VPGPFE601N) | 1.00E-08 | 94 |
| Contig00154 | PREDICTED: *Vitis vinifera* NEDD8-activating enzyme E1 catalytic subunit-like (LOC100256207), mRNA [gi|225432555|XM_002280892.1](http://www.ncbi.nlm.nih.gov/nucleotide/225432555?report=genbank&log$=nucltop&blast_rank=3&RID=4VBY80DF01N) | | 4.00E-18 | | 87 | | | PREDICTED: NEDD8-activating enzyme E1 catalytic subunit-like [*Glycine max*] [XP_003549989.1](http://www.ncbi.nlm.nih.gov/protein/356563478?report=genbank&log$=prottop&blast_rank=1&RID=4VS984W8014) | 2.00E-12 | 84 |
| HSZW1U101A1ZEV | *Arabidopsis thaliana* putative ubiquitin extension protein UBQ1 (At3g52590) mRNA, complete cds [AY059080.1](http://www.ncbi.nlm.nih.gov/nucleotide/16323383?report=genbank&log$=nucltop&blast_rank=7&RID=4XJTXFGF014) | | 2.00E-14 | | 95 | | | Ubiquitin [*Medicago truncatula*] [XP_003602319.1](http://www.ncbi.nlm.nih.gov/protein/357464075?report=genbank&log$=prottop&blast_rank=8&RID=50SAP4J801N) | 0.001 | 100 |
| HSZW1U101A2BAG | *Arabidopsis thaliana* 26S proteasome non-ATPase regulatory subunit [NM_105127.3](http://www.ncbi.nlm.nih.gov/nucleotide/145337165?report=genbank&log$=nucltop&blast_rank=3&RID=4YGNKCWZ01N) | | 4.00E-11 | | 79 | | | 26S proteasome subunit RPN12 [*Arabidopsis thaliana*] [AAP86674.1](http://www.ncbi.nlm.nih.gov/protein/32700048?report=genbank&log$=prottop&blast_rank=3&RID=50HGN0CJ01N) | 1.00E-08 | 66 |
| HSZW1U101A1UQD | *Capsicum annuum* ubiquitin-conjugating protein mRNA, complete cds [AY486137.1](http://www.ncbi.nlm.nih.gov/nucleotide/40287567?report=genbank&log$=nucltop&blast_rank=2&RID=4X8CKEMZ014) | | 2.00E-12 | | 77 | | | PREDICTED protein [*Hordeum vulgare* subsp*. vulgare*] [BAK06849.1](http://www.ncbi.nlm.nih.gov/protein/326513218?report=genbank&log$=prottop&blast_rank=1&RID=4ZFDV1EN01N) | 0.001 | 46 |
| HSZW1U101A9CPU | *Glycine max* 26S proteasome non-ATPase regulatory subunit RPN12A-like (LOC100785835), mRNA [NM_001254242.1](http://www.ncbi.nlm.nih.gov/nucleotide/359807665?report=genbank&log$=nucltop&blast_rank=4&RID=4XG8H2WB01N) | | 2.00E-06 | | 78 | | | 26S proteasome non-ATPase regulatory subunit RPN12A [*Arabidopsis thaliana*] [NP_176633.1](http://www.ncbi.nlm.nih.gov/protein/15217661?report=genbank&log$=prottop&blast_rank=2&RID=50ZAUAC7014) | 1.00E-06 | 72 |
| **STRESS-RELATED RESPONSES** | | | | | | | | | | |
| Contig00048 | *Nicotiana tabacum* NtHsp90 mRNA for Heat shock protein 90, complete cds [gi|392465168|AB689674.1](http://www.ncbi.nlm.nih.gov/nucleotide/392465168?report=genbank&log$=nucltop&blast_rank=2&RID=4V8C796R01N) | | 4.00E-91 | | 90 | | | HSP80 [*Populus alba*] [AAN87002.1](http://www.ncbi.nlm.nih.gov/protein/27362887?report=genbank&log$=prottop&blast_rank=1&RID=4VKW2CX701N) | 2.00E-24 | 89 |
| Contig0090 | *Nicotiana glauca* X *Nicotiana langsdorffii* mRNA for tumor-related protein, partial sequence, clone:tid793 [gi|454202|D26466.1](http://www.ncbi.nlm.nih.gov/nucleotide/454202?report=genbank&log$=nucltop&blast_rank=1&RID=4VBT91G501N) | | 1.00E-38 | | 87 | | | Putative protein [*Arabidopsis thaliana*]  [CAA20575.1](http://www.ncbi.nlm.nih.gov/protein/3549664?report=genbank&log$=prottop&blast_rank=1&RID=4VPHA3G5014) | 9.00E-05 | 38 |
| Contig00118 | *Nicotiana tabacum* partial mRNA for putative glutathione S-transferase (GST1 gene), clone EBR-52 [gi|68687852|AJ937852.1](http://www.ncbi.nlm.nih.gov/nucleotide/68687852?report=genbank&log$=nucltop&blast_rank=1&RID=4VBUZAEZ01N) | | 1.00E-57 | | 87 | | | Putative glutathione S-transferase [*Nicotiana tabacum*]  [CAI78906.1](http://www.ncbi.nlm.nih.gov/protein/68687853?report=genbank&log$=prottop&blast_rank=1&RID=4VS8EAA3016) | 8.00E-14 | 86 |
| Contig00053 | *Solanum lycopersicum* cultivar Rio Grande PtoR Hop-interacting protein THI111 mRNA, complete cds [gi|365222919|GQ261256.1](http://www.ncbi.nlm.nih.gov/nucleotide/365222919?report=genbank&log$=nucltop&blast_rank=3&RID=4V8C796R01N) | | 1.00E-67 | | 90 | | | Hop-interacting protein THI111 [*Solanum lycopersicum*] [AEW69812.1](http://www.ncbi.nlm.nih.gov/protein/365222920?report=genbank&log$=prottop&blast_rank=1&RID=4VKWP2XC01N) | 5.00E-31 | 86 |
| HSZW1U101BSQQM | *Arabidopsis thaliana* universal stress protein (USP) family protein (AT3G53990) mRNA, complete cds [NM_115259.4](http://www.ncbi.nlm.nih.gov/nucleotide/186511038?report=genbank&log$=nucltop&blast_rank=5&RID=4XJSDK4G014) | | 5.00E-31 | | 79 | | | Universal stress protein (USP) family protein [*Arabidopsis thaliana*] [NP_974427.1](http://www.ncbi.nlm.nih.gov/protein/42572663?report=genbank&log$=prottop&blast_rank=5&RID=50SCHD4B014) | 8.00E-29 | 80 |
| HSZW1U101A0XGV | *Pyrus pyrifolia* var*. culta* TIP 1 mRNA for putative tonoplast intrinsic protein 1, partial cds [AB721408.1](http://www.ncbi.nlm.nih.gov/nucleotide/388281851?report=genbank&log$=nucltop&blast_rank=15&RID=4W4DAS5001N) | | 7.00E-04 | | 100 | | | Putative tonoplast intrinsic protein 1, partial [*Pyrus pyrifolia* var*. culta*] [BAM15884.1](http://www.ncbi.nlm.nih.gov/protein/388281852?report=genbank&log$=prottop&blast_rank=2&RID=4YVRY23W016) | 0.028 | 67 |
| HSZW1U101BYM2D | *Rosa* hybrid cultivar cytosolic class II small heat-shock protein HSP17.5 (hsp17.5) mRNA, complete cds [EF157600.1](http://www.ncbi.nlm.nih.gov/nucleotide/140083604?report=genbank&log$=nucltop&blast_rank=1&RID=4W358UJA01N) | | 1.00E-08 | | 83 | | | Class II small heat shock protein Le-HSP17.6 [*Arachis hypogaea*] [ACF74271.1](http://www.ncbi.nlm.nih.gov/protein/194466081?report=genbank&log$=prottop&blast_rank=1&RID=4YDWAEUZ014) | 1.00E-07 | 77 |
| HSZW1U101A4ONF | PREDICTED: *Vitisvinifera* serine hydroxymethyltransferase, mitochondrial-like (LOC100245411), mRNA [XM_002285569.2](http://www.ncbi.nlm.nih.gov/nucleotide/359492974?report=genbank&log$=nucltop&blast_rank=2&RID=4YJ4ARR9016) | | 7.00E-15 | | 87 | | | Glycine/serine hydroxymethyltransferasereductase, partial [*Olea europaea*] [ABS72016.1](http://www.ncbi.nlm.nih.gov/protein/154257305?report=genbank&log$=prottop&blast_rank=1&RID=50FAZTKP01N) | 7.00E-05 | 66 |
| **DEFENSE RESPONSES** | | | | | | | | | | |
| Contig00026 | *Nicotiana tabacum* mRNA for pre-pro-cysteine proteinase [gi|19850|Z13964.1](http://www.ncbi.nlm.nih.gov/nucleotide/19850?report=genbank&log$=nucltop&blast_rank=1&RID=4V88EKR601N) | | 8.00E-159 | | 98 | | | Putative preprocysteine proteinase [*Nicotiana tabacum*] [CAB44983.1](http://www.ncbi.nlm.nih.gov/protein/5051468?report=genbank&log$=prottop&blast_rank=1&RID=4VMSFR6B016) | 2.00E-20 | 95 |
| Contig00045 | *Nicotiana tabacum* mRNA for thioredoxin peroxidase [gi|21912926|AJ309009.2](http://www.ncbi.nlm.nih.gov/nucleotide/21912926?report=genbank&log$=nucltop&blast_rank=1&RID=4V8C796R01N) | | 1.00E-104 | | 100 | | | Thioredoxin peroxidase [*Nicotiana tabacum*] [CAC84143.2](http://www.ncbi.nlm.nih.gov/protein/21912927?report=genbank&log$=prottop&blast_rank=1&RID=4VKW2CX701N) | 5.00E-14 | 100 |
| Contig00056 | *Nicotiana tabacum* partial mRNA for putative stress related chitinase (cht STR1 gene), clone CHO3E10 [gi|62719020|AJ880385.1](http://www.ncbi.nlm.nih.gov/nucleotide/62719020?report=genbank&log$=nucltop&blast_rank=1&RID=4V8FEUY701N) | | 1.00E-117 | | 98 | | | Hypothetical protein OsI_*26311* [*Oryza sativa Indica Group*] [EAZ04169.1](http://www.ncbi.nlm.nih.gov/protein/125558633?report=genbank&log$=prottop&blast_rank=1&RID=4VKWP2XC01N) | 7.00E-05 | 57 |
| Contig00093 | *Nicotiana tabacum* Avr9/Cf-9 rapidly elicited protein 261 (ACRE261) mRNA, partial cds [gi|56544481|AY775047.1](http://www.ncbi.nlm.nih.gov/nucleotide/56544481?report=genbank&log$=nucltop&blast_rank=2&RID=4VBT91G501N) | | 3.00E-40 | | 83 | | | Avr9/Cf-9 rapidly elicited protein 261, partial [*Nicotiana tabacum*] [AAV92906.1](http://www.ncbi.nlm.nih.gov/protein/56544482?report=genbank&log$=prottop&blast_rank=1&RID=4VPJUZEH014) | 1.00E-13 | 79 |
| Contig00157 | *Ricinus communis* nonsense-mediated mRNA decay protein, putative, [gi|255575789|XM_002528748.1](http://www.ncbi.nlm.nih.gov/nucleotide/255575789?report=genbank&log$=nucltop&blast_rank=2&RID=4VBY80DF01N) | | 1.00E-10 | | 91 | | | Hypothetical protein SORBIDRAFT_02g029430 [*Sorghum bicolor*] [XP_002462642.1](http://www.ncbi.nlm.nih.gov/protein/242049796?report=genbank&log$=prottop&blast_rank=1&RID=4VS984W8014) | 0.002 | 43 |
| HSZW1U101BMETO | PREDICTED: *Glycine max* non-specific lipid-transfer protein 2-like (LOC100794984), mRNA XM_003524518.1 | | 0.54 | | 92 | | | Accelerated cell death 1 [*Arabidopsis thaliana*] AAR05797.1 | 6.00E-11 | 58 |
| HSZW1U101A7RSU | *Glycine max* programmed cell death protein 2-like (LOC100818325), mRNA [NM_001254111.1](http://www.ncbi.nlm.nih.gov/nucleotide/359806730?report=genbank&log$=nucltop&blast_rank=25&RID=4XCDTXDB01N) | | 0.004 | | 86 | | | PREDICTED: uncharacterized protein LOC100806570 [*Glycine max*][XP_003516905.1](http://www.ncbi.nlm.nih.gov/protein/356496094?report=genbank&log$=prottop&blast_rank=5&RID=50GATHMW01N) | 8.00E-04 | 73 |
| HSZW1U101A3L23 | *Nicotiana glutinosa* biotic cell death-associated protein (CDM1) mRNA, complete cds [AF208022.1](http://www.ncbi.nlm.nih.gov/nucleotide/6538777?report=genbank&log$=nucltop&blast_rank=1&RID=4XJRU29501N) | | 8.00E-37 | | 75 | | | Biotic cell death-associated protein [*Nicotiana glutinosa*][AAF15902.1](http://www.ncbi.nlm.nih.gov/protein/6538778?report=genbank&log$=prottop&blast_rank=1&RID=50SD4MCT014) | 4.00E-13 | 64 |
| HSZW1U101A9XP5 | *Nicotiana tabacum*pheophorbide A oxygenase 1 mRNA, complete cds [ABY19384.1](http://www.ncbi.nlm.nih.gov/protein/162568916?report=genbank&log$=prottop&blast_rank=1&RID=50DMPMP001N) | | 4.00E-109 | | 99 | | | Pheophorbide A oxygenase 1 [*Nicotiana tabacum*][ABY19384.1](http://www.ncbi.nlm.nih.gov/protein/162568916?report=genbank&log$=prottop&blast_rank=1&RID=50DMPMP001N) | 2.00E-17 | 97 |
| HSZW1U101BMBLT | *Nicotiana tabacum*pheophorbide A oxygenase 1 mRNA, complete cds [EU294211.1](http://www.ncbi.nlm.nih.gov/nucleotide/162568915?report=genbank&log$=nucltop&blast_rank=1&RID=4X9W3TKH014) | | 5.00E-93 | | 99 | | | Pheophorbide A oxygenase 1 [*Nicotiana tabacum*] [ABY19384.1](http://www.ncbi.nlm.nih.gov/protein/162568916?report=genbank&log$=prottop&blast_rank=1&RID=50DY6BT301N) | 2.00E-18 | 100 |
| HSZW1U101BZ7E8 | Osmotin=pathogenesis-related protein homolog [*Nicotiana tabacum*=tobacco, cv Samsun nn, floral bud day 7 explant, mRNA Partial, 928 nt] [S44889.1](http://www.ncbi.nlm.nih.gov/nucleotide/256134?report=genbank&log$=nucltop&blast_rank=2&RID=4W9MVG6Y014) | | 4.00E-07 | | 100 | | | CC-NBS-LRR resistance protein [*Medicago truncatula*] [XP_003626885.1](http://www.ncbi.nlm.nih.gov/protein/357513193?report=genbank&log$=prottop&blast_rank=2&RID=4Z04UJ45014) | 0.002 | 56 |
| **TRANSCRIPTION AND PROTEIN SYNTHESIS** | | | | | | | | | | |
| Contig00014 | *Jatropha curcas* putative small nuclear ribonucleoprotein polypeptide[gi|282848217|FJ899653.1](http://www.ncbi.nlm.nih.gov/nucleotide/282848217?report=genbank&log$=nucltop&blast_rank=6&RID=4V86XG1001N) | | 2.00E-33 | | 81 | | | PREDICTED: small nuclear ribonucleoprotein G [*Glycine max*] [XP_003537685.1](http://www.ncbi.nlm.nih.gov/protein/356538387?report=genbank&log$=prottop&blast_rank=2&RID=4VKTA5WY01N) | 2.00E-29 | 87 |
| Contig00020 | *Nicotiana tabacum* EF-1-alpha-related GTP-binding protein (SUP1) mRNA, complete cds [gi|1009231|L38828.1](http://www.ncbi.nlm.nih.gov/nucleotide/1009231?report=genbank&log$=nucltop&blast_rank=1&RID=4V88EKR601N) | | 4.00E-144 | | 96 | | | *Nicotiana tabacum* EF-1-alpha-related GTP-binding protein (SUP1) mRNA, complete cds [L38828.1](http://www.ncbi.nlm.nih.gov/nucleotide/1009231?report=genbank&log$=nucltop&blast_rank=1&RID=5DME42B001N) | 2.00E-47 | 96 |
| Contig00019 | *Nicotiana tabacum* clone 7 poly(A)-binding protein (PABP) mRNA, partial cds [gi|7673358|AF190657.1](http://www.ncbi.nlm.nih.gov/nucleotide/7673358?report=genbank&log$=nucltop&blast_rank=1&RID=4V88EKR601N) | | 3.00E-151 | | 98 | | | poly(A)-binding protein [*Nicotiana tabacum*] [AAF66825.1](http://www.ncbi.nlm.nih.gov/protein/7673359?report=genbank&log$=prottop&blast_rank=1&RID=4VKTA5WY01N) | 4.00E-18 | 98 |
| Contig00060 | *Nicotiana tabacum* putative RNA binding protein (QRRBP-1) mRNA, partial cds [gi|2708531|AF029351.1](http://www.ncbi.nlm.nih.gov/nucleotide/2708531?report=genbank&log$=nucltop&blast_rank=1&RID=4V8FEUY701N) | | 3.00E-105 | | 93 | | | putative RNA binding protein [*Nicotiana tabacum*][AAB92518.1](http://www.ncbi.nlm.nih.gov/protein/2708532?report=genbank&log$=prottop&blast_rank=2&RID=4VKWP2XC01N) | 0.001 | 100 |
| Contig 00001 | *Nicotiana tabacum* cyclophilin-like (CYP1) mRNA, complete sequence [gi|46404795|AY368274.1](http://www.ncbi.nlm.nih.gov/nucleotide/46404795?report=genbank&log$=nucltop&blast_rank=1&RID=4V861KCC01N) | | 0 | | 97 | | | Cyclophilin [*Capsicum annuum*] [ACB05668.1](http://www.ncbi.nlm.nih.gov/protein/169930141?report=genbank&log$=prottop&blast_rank=1&RID=4VKKKDU501N) | 1.00E-67 | 97 |
| Contig00041 | PREDICTED:*Vitis vinifera*protein translation factor SUI1 homolog-like [gi|225461084|XM_002281836.1](http://www.ncbi.nlm.nih.gov/nucleotide/225461084?report=genbank&log$=nucltop&blast_rank=18&RID=4V8AJSEW01N) | | 3.00E-17 | | 86 | | | Translation factor Sui1-like protein [*Medicago truncatula*] [XP_003596650.1](http://www.ncbi.nlm.nih.gov/protein/357452747?report=genbank&log$=prottop&blast_rank=5&RID=4VKUYFRB01N) | 1.00E-11 | 100 |
| HSZW1U101BOPHX | *Arabidopsis thaliana* thioredoxin M2 (ATHM2) mRNA, complete cds [NM_001160731.1](http://www.ncbi.nlm.nih.gov/nucleotide/238480209?report=genbank&log$=nucltop&blast_rank=7&RID=4XDHZ5DV014) | | 1.00E-18 | | 81 | | | Thioredoxin m(mitochondrial)-type, putative [*Ricinus communis*] [XP_002520230.1](http://www.ncbi.nlm.nih.gov/protein/255558409?report=genbank&log$=prottop&blast_rank=1&RID=50GEVAPR01N) | 2.00E-15 | 86 |
| HSZW1U101BWESW | *Freesia refracta* putative nucleic acid binding protein mRNA, partial cds [GU323320.1](http://www.ncbi.nlm.nih.gov/nucleotide/284810442?report=genbank&log$=nucltop&blast_rank=10&RID=4Y4T3RRT016) | | 4.00E-08 | | 83 | | | Nucleic acid binding protein, putative [*Ricinus communis*] [XP_002521418.1](http://www.ncbi.nlm.nih.gov/protein/255560810?report=genbank&log$=prottop&blast_rank=5&RID=50RGX9Y4014) | 1.00E-04 | 92 |
| HSZW1U101BYFG3 | *Medicago truncatula* Peptidyl-prolyl cis-trans isomerase (MTR_4g075290) mRNA, complete cds [XM_003607224.1](http://www.ncbi.nlm.nih.gov/nucleotide/357473974?report=genbank&log$=nucltop&blast_rank=18&RID=4XJRU29501N) | | 4.00E-09 | | 88 | | | Peptidyl-prolyl *cis*-*trans* isomerase [P35627.1](http://www.ncbi.nlm.nih.gov/protein/544125?report=genbank&log$=prottop&blast_rank=5&RID=50SD4MCT014) | 2.00E-04 | 64 |
| HSZW1U101BWFKF | PREDICTED: *Vitis vinifera* DNA-directed RNA polymerases I, II, and III subunit RPABC4-like (LOC100252452), mRNA [XM_002272018.2](http://www.ncbi.nlm.nih.gov/nucleotide/359490036?report=genbank&log$=nucltop&blast_rank=2&RID=4W4CC8GT01N) | | 3.00E-33 | | 85 | | | PREDICTED: DNA-directed RNA polymerases I, II, and III subunit RPABC4-like [*Vitis vinifera*] [XP_002267800.1](http://www.ncbi.nlm.nih.gov/protein/225466239?report=genbank&log$=prottop&blast_rank=1&RID=4YVRY23W016) | 2.00E-28 | 98 |
| **METABOLISM AND ENERGY** | | | | | | | | | | |
| Contig 00003 | *Camellia sinensis* clone U10BcDNA 3161 acyl-CoA-binding protein mRNA, complete cds [gi|330318653|HM003274.1](http://www.ncbi.nlm.nih.gov/nucleotide/330318653?report=genbank&log$=nucltop&blast_rank=6&RID=4V861KCC01N) | | 1.00E-70 | | 86 | | | Acyl-CoA-binding protein [*Panax ginseng*] [BAB85987.1](http://www.ncbi.nlm.nih.gov/protein/19352190?report=genbank&log$=prottop&blast_rank=1&RID=4VKSK9DZ01N) | 4.00E-44 | 85 |
| Contig00021 | *Oryza glumipatula* EPSPs, rps20 genes for 5-enolpyruvylshikimate-3-phosphate synthase, ribosomal protein small subunit 20, partial sequences, cultivar:W1185 [gi|46559339|AB124888.1](http://www.ncbi.nlm.nih.gov/nucleotide/46559339?report=genbank&log$=nucltop&blast_rank=9&RID=4V88EKR601N) | | 4.00E-29 | | 88 | | | PREDICTEDprotein [*Populus trichocarpa*] [XP_002318875.1](http://www.ncbi.nlm.nih.gov/protein/224122592?report=genbank&log$=prottop&blast_rank=1&RID=4VKTA5WY01N) | 1.00E-15 | 97 |
| Contig00057 | *Nicotiana tabacum* mRNA for citrate synthase [gi|1556428|X84226.1](http://www.ncbi.nlm.nih.gov/nucleotide/1556428?report=genbank&log$=nucltop&blast_rank=1&RID=4V8FEUY701N) | | 5.00E-65 | | 91 | | | Pyruvate decarboxylase isozyme[*Medicago truncatula*] [XP_003623316.1](http://www.ncbi.nlm.nih.gov/protein/357506055?report=genbank&log$=prottop&blast_rank=5&RID=4VKWP2XC01N) | 4.00E-04 | 50 |
| Contig00022 | *Petunia hybrida* mRNA for triosephosphate isomerase [gi|602589|X83227.1](http://www.ncbi.nlm.nih.gov/nucleotide/602589?report=genbank&log$=nucltop&blast_rank=1&RID=4V88EKR601N) | | 8.00E-121 | | 87 | | | Triose phosphate isomerase-like protein type II [*Dimocarpus longan*] [ACY66804.1](http://www.ncbi.nlm.nih.gov/protein/262410515?report=genbank&log$=prottop&blast_rank=1&RID=4VKTA5WY01N) | 1.00E-37 | 94 |
| Contig00033 | *Solanum tuberosum*transaldolase (PotTal1) mRNA, complete cds [gi|2078349|U95923.1](http://www.ncbi.nlm.nih.gov/nucleotide/2078349?report=genbank&log$=nucltop&blast_rank=1&RID=4V89D0SK01N) | | 8.00E-70 | | 81 | | | Transaldolase [*Solanum lycopersicum*] [AAP83926.1](http://www.ncbi.nlm.nih.gov/protein/32481059?report=genbank&log$=prottop&blast_rank=1&RID=4VKUYFRB01N) | 1.00E-21 | 84 |
| Contig00011 | PREDICTED: *Vitis vinifera* diaminopimelate decarboxylase 2, chloroplastic-like, transcript variant 2 (LOC100247076), mRNA [gi|225428409|XM_002283680.1](http://www.ncbi.nlm.nih.gov/nucleotide/225428409?report=genbank&log$=nucltop&blast_rank=2&RID=4V86XG1001N) | | 1.00E-67 | | 84 | | | Diaminopimelate decarboxylase [*Zea mays*] [ACG33840.1](http://www.ncbi.nlm.nih.gov/protein/195624020?report=genbank&log$=prottop&blast_rank=1&RID=4VKTA5WY01N) | 2.00E-24 | 95 |
| HSZW1U101A18CH | *Medicago truncatula* Cytochrome c oxidase subunit (MTR_1g006950) [XM_003588363.1](http://www.ncbi.nlm.nih.gov/nucleotide/357436270?report=genbank&log$=nucltop&blast_rank=26&RID=4YKFSH3T014) | | 1.00E-16 | | 98 | | | Cytochrome c oxidase subunit 1 [*Oryza rufipogon*] [YP_003433868.1](http://www.ncbi.nlm.nih.gov/protein/289065057?report=genbank&log$=prottop&blast_rank=1&RID=50E29XZ501N) | 7.00E-06 | 89 |
| HSZW1U101A617E | *Nicotiana tabacum* Nt-5beta-POR-A mRNA for progesterone 5beta reductase-A, complete cds [AB488494.1](http://www.ncbi.nlm.nih.gov/nucleotide/226235455?report=genbank&log$=nucltop&blast_rank=1&RID=4YFX6ARM01N) | | 6.00E-49 | | 98 | | | Progesterone 5beta reductase-A [*Nicotianatabacum*] [BAH47640.1](http://www.ncbi.nlm.nih.gov/protein/226235456?report=genbank&log$=prottop&blast_rank=1&RID=50HGN0CJ01N) | 3.00E-10 | 100 |
| HSZW1U101A0KHO | *Solanumtuberosum* mRNA for NADH-ubiquinone oxidoreductase subunit [X96671.1](http://www.ncbi.nlm.nih.gov/nucleotide/1235606?report=genbank&log$=nucltop&blast_rank=1&RID=4YKGC60W014) | | 1.00E-23 | | 78 | | | NADH ubiquinone oxidoreductase PSST subunit [*Lupinus luteus*] [AAF01037.1](http://www.ncbi.nlm.nih.gov/protein/6007781?report=genbank&log$=prottop&blast_rank=1&RID=50E1DUEC01N) | 2.00E-07 | 84 |
| HSZW1U101BUD3N | *Nicotiana tabacum* NtGT2 mRNA for glucosyltransferase, complete cds [AB072919.1](http://www.ncbi.nlm.nih.gov/nucleotide/20146092?report=genbank&log$=nucltop&blast_rank=1&RID=4XG6GYGJ014) | | 2.00E-51 | | 94 | | | Glucosyltransferase [*Nicotiana tabacum*] [BAB88935.1](http://www.ncbi.nlm.nih.gov/protein/20146093?report=genbank&log$=prottop&blast_rank=1&RID=50ZA40UM01N) | 5.00E-18 | 73 |
| **REGULATION** | | | | | | | | | | |
| Contig00127 | *Nicotiana tabacum* NtMBF1a mRNA for putative multiprotein bridging factor 1, complete cds [gi|20086363|AB072698.1](http://www.ncbi.nlm.nih.gov/nucleotide/20086363?report=genbank&log$=nucltop&blast_rank=1&RID=4VBVXV4101N) | | 2.00E-36 | | 89 | | | Hypothetical protein [*Oryza sativa Japonica* Group] [BAC79189.1](http://www.ncbi.nlm.nih.gov/protein/32526666?report=genbank&log$=prottop&blast_rank=1&RID=4VS8EAA3016) | 6.00E-04 | 52 |
| HSZW1U101BN50Q | *Arabidopsis thaliana* telomere repeat binding factor 3 (TRB3) gene, complete cds [U83838.1](http://www.ncbi.nlm.nih.gov/nucleotide/18481423?report=genbank&log$=nucltop&blast_rank=57&RID=4YFW5Z5E014) | | 9.00E-15 | | 74 | | | ADP-ribosylation factor-like 8b [*Nicotiana tabacum*] [BAL44263.1](http://www.ncbi.nlm.nih.gov/protein/371501272?report=genbank&log$=prottop&blast_rank=1&RID=50K7EHT5016) | 2.00E-27 | 100 |
| HSZW1U101BSD87 | *Nicotiana attenuata* SGT1 mRNA, complete cds [GU265726.1](http://www.ncbi.nlm.nih.gov/nucleotide/315307973?report=genbank&log$=nucltop&blast_rank=1&RID=4Y4S4G3Z014) | | 2.00E-09 | | 98 | | | SGT1 [*Nicotiana attenuata*] [ADU04390.1](http://www.ncbi.nlm.nih.gov/protein/315307974?report=genbank&log$=prottop&blast_rank=1&RID=50RJ7K0C016) | 5.00E-05 | 87 |
| HSZW1U101BUK1L | *Nicotiana tabacum* NtARL8b mRNA for ADP-ribosylation factor-like 8b, complete cds [AB644218.1](http://www.ncbi.nlm.nih.gov/nucleotide/371501271?report=genbank&log$=nucltop&blast_rank=1&RID=4XG7V82D01N) | | 8.00E-42 | | 100 | | | ADP-ribosylation factor-like 8b [*Nicotiana tabacum*] [BAL44263.1](http://www.ncbi.nlm.nih.gov/protein/371501272?report=genbank&log$=prottop&blast_rank=1&RID=50ZA40UM01N) | 2.00E-15 | 100 |
| HSZW1U101BVCN6 | *Nicotiana tabacum* putative ROX1 mRNA, complete cds[DQ866831.1](http://www.ncbi.nlm.nih.gov/nucleotide/110826034?report=genbank&log$=nucltop&blast_rank=1&RID=4W1KMW5E01N) | | 1.00E-103 | | 99 | | | Putative ROX1 [*Nicotiana tabacum*] [ABH01084.1](http://www.ncbi.nlm.nih.gov/protein/110826035?report=genbank&log$=prottop&blast_rank=1&RID=4Y6F221B014) | 8.00E-24 | 84 |
| HSZW1U101BV2W9 | *Nicotiana sylvestris* mRNA for cytokinin binding protein CBP57, complete cds [D16139.1](http://www.ncbi.nlm.nih.gov/nucleotide/441218?report=genbank&log$=nucltop&blast_rank=2&RID=4Y9JV39Z016) | | 2.00E-49 | | 93 | | | Cytokinin binding protein CBP57 [*Nicotiana sylvestris*] [BAA03710.1](http://www.ncbi.nlm.nih.gov/protein/2160322?report=genbank&log$=prottop&blast_rank=2&RID=50PHYGHK014) | 1.00E-19 | 92 |
| HSZW1U101BW4OV | *Solanum lycopersicum* ripening regulated protein DDTFR19 (DDTFR19), mRNA [NM_001247550.1](http://www.ncbi.nlm.nih.gov/nucleotide/350535970?report=genbank&log$=nucltop&blast_rank=8&RID=4X8B6SSM014) | | 5.00E-13 | | 91 | | | Ripening regulated protein DDTFR19 [*Solanum lycopersicum*] [NP_001234479.1](http://www.ncbi.nlm.nih.gov/protein/350535971?report=genbank&log$=prottop&blast_rank=2&RID=4ZFCC07C01N) | 2.00E-08 | 100 |
| **TRANSPORT** | | | | | | | | | | |
| Contig00042 | *Arabidopsis thaliana* putative copper transport protein (AT5G23760) mRNA, complete cds [gi|145358359|NM_122281.3](http://www.ncbi.nlm.nih.gov/nucleotide/145358359?report=genbank&log$=nucltop&blast_rank=6&RID=4V8AJSEW01N) | | 3.00E-50 | | 79 | | | Putative metal ion-binding protein [*Linum usitatissimum*] [AFN53686.1](http://www.ncbi.nlm.nih.gov/protein/395146532?report=genbank&log$=prottop&blast_rank=1&RID=4VKW2CX701N) | 8.00E-29 | 87 |
| Contig00038 | *Medicago truncatula* Nuclear transport factor (MTR_3g085070) mRNA, complete cds [gi|357462952|XM_003601710.1](http://www.ncbi.nlm.nih.gov/nucleotide/357462952?report=genbank&log$=nucltop&blast_rank=6&RID=4V8AJSEW01N) | | 3.00E-12 | | 89 | | | PREDICTED: LOW QUALITY PROTEIN: nuclear transport factor 2-like [*Glycine max*] [XP_003553218.1](http://www.ncbi.nlm.nih.gov/protein/356570078?report=genbank&log$=prottop&blast_rank=1&RID=4VKUYFRB01N) | 8.00E-06 | 100 |
| Contig00034 | *Nicotiana tabacum* NtMATE2 mRNA for multi antimicrobial extrusion family protein, complete cds [gi|126363775|AB286962.1](http://www.ncbi.nlm.nih.gov/nucleotide/126363775?report=genbank&log$=nucltop&blast_rank=1&RID=4V89D0SK01N) | | 1.00E-149 | | 98 | | | Multi antimicrobial extrusion family protein [*Nicotiana tabacum*] [BAF47752.1](http://www.ncbi.nlm.nih.gov/protein/126363776?report=genbank&log$=prottop&blast_rank=1&RID=4VKUYFRB01N) | 2.00E-13 | 100 |
| Contig00055 | *Solanum tuberosum* clone 021G11 translocon-associated protein beta family protein-like mRNA, complete cds [gi|82623434|DQ241861.1](http://www.ncbi.nlm.nih.gov/nucleotide/82623434?report=genbank&log$=nucltop&blast_rank=1&RID=4V8FEUY701N) | | 2.00E-81 | | 93 | | | Translocon-associated protein beta family protein-like [*Solanum tuberosum*] [ABB87132.1](http://www.ncbi.nlm.nih.gov/protein/82623435?report=genbank&log$=prottop&blast_rank=1&RID=4VKWP2XC01N) | 7.00E-30 | 92 |
| HSZW1U101BXHUQ | *Arabidopsis thaliana* Endoplasmic reticulum vesicle transporter protein (AT1G36050) mRNA, [NM_103301.6](http://www.ncbi.nlm.nih.gov/nucleotide/240254209?report=genbank&log$=nucltop&blast_rank=2&RID=4Y9ME1UR01N) | | 5.00E-10 | | 86 | | | Endoplasmic reticulum vesicle transporter protein [*Arabidopsis thaliana*] [NP_564467.5](http://www.ncbi.nlm.nih.gov/protein/240254210?report=genbank&log$=prottop&blast_rank=2&RID=50PGJA48014) | 4.00E-09 | 86 |
| HSZW1U101BY2Q8 | *PREDICTED: Glycine max ABC transporter B family member 25-like (LOC100810510), miscRNA* [*XR_137452.1*](http://www.ncbi.nlm.nih.gov/nucleotide/356564942?report=genbank&log$=nucltop&blast_rank=6&RID=4XHDY00C016) | | 2.00E-15 | | 85 | | | ABC transporter family protein [Populus trichocarpa]  [XP_002324121.1](http://www.ncbi.nlm.nih.gov/protein/224141527?report=genbank&log$=prottop&blast_rank=1&RID=50UR50GD01N) | 2.00E-08 | 55 |
| HSZW1U101A38AJ | *PREDICTED: Vitis vinifera endoplasmic reticulum-Golgi intermediate compartment protein 3-like (LOC100267365), mRNA* [*XM_002264608.2*](http://www.ncbi.nlm.nih.gov/nucleotide/359485743?report=genbank&log$=nucltop&blast_rank=8&RID=4XJUPZSA014) | | 9.00E-04 | | 83 | | | Endoplasmic reticulum-Golgi intermediate compartment protein [Medicago truncatula]  [XP_003615024.1](http://www.ncbi.nlm.nih.gov/protein/357489473?report=genbank&log$=prottop&blast_rank=2&RID=50SA2FEN014) | 7.00E-06 | 89 |
| **CELL WALL-RELATED** | | | | | | | | | | |
| Contig 00004 | *Arabidopsis thaliana* putative proline-rich cell wall protein (At1g62500) mRNA, complete [gi|19310692|AY079346.1](http://www.ncbi.nlm.nih.gov/nucleotide/19310692?report=genbank&log$=nucltop&blast_rank=5&RID=4V861KCC01N) | | 3.00E-20 | | 77 | | | Proline rich protein [*Medicago truncatula*]  [XP_003608742.1](http://www.ncbi.nlm.nih.gov/protein/357476913?report=genbank&log$=prottop&blast_rank=2&RID=4VKSK9DZ01N) | 4.00E-12 | 82 |
| Contig 00006 | *Nicotiana tabacum* mRNA for P-rich protein EIG-I30, complete cds  [gi|10798751|AB041516.1](http://www.ncbi.nlm.nih.gov/nucleotide/10798751?report=genbank&log$=nucltop&blast_rank=1&RID=4V861KCC01N) | | 1.00E-178 | | 100 | | | P-rich protein EIG-I30[*Nicotiana tabacum*]  [BAB16428.1](http://www.ncbi.nlm.nih.gov/protein/10798752?report=genbank&log$=prottop&blast_rank=1&RID=4VKSK9DZ01N) | 7.00E-14 | 100 |
| Contig00100 | *Pyrus pyrifolia* var. *culta* mRNA for putative annexin, partial cds  [gi|388281855|AB721410.1](http://www.ncbi.nlm.nih.gov/nucleotide/388281855?report=genbank&log$=nucltop&blast_rank=2&RID=4VBU3ETS01N) | | 2.00E-04 | | 94 | | | PREDICTED: transmembrane protein 50 homolog [*Vitis vinifera*] [XP_002285830.1](http://www.ncbi.nlm.nih.gov/protein/225459447?report=genbank&log$=prottop&blast_rank=1&RID=4VPJUZEH014) | 8.00E-04 | 35 |
| Contig00144 | *Nicotiana tabacum* gene for extensin, complete cds  [gi|505143|D13951.1](http://www.ncbi.nlm.nih.gov/nucleotide/505143?report=genbank&log$=nucltop&blast_rank=1&RID=4VBWU89T01N) | | 2.00E-73 | | 100 | | | Extensin - tomato (fragment)  [S49760](http://www.ncbi.nlm.nih.gov/protein/1076582?report=genbank&log$=prottop&blast_rank=1&RID=4VS8EAA3016) | 2.00E-08 | 69 |
| HSZW1U101A1YZN | *Arabidopsis thaliana* glycine-rich protein (AT4G21620) mRNA, complete cds [NM_001203859.1](http://www.ncbi.nlm.nih.gov/nucleotide/334186780?report=genbank&log$=nucltop&blast_rank=10&RID=4XES38XB016) | | 2.00E-08 | | 75 | | | Glycine-rich protein [*Arabidopsis thaliana*]  [NP_001190788.1](http://www.ncbi.nlm.nih.gov/protein/334186781?report=genbank&log$=prottop&blast_rank=1&RID=50J5YSW301N) | 8.00E-09 | 94 |
| HSZW1U101BU0OC | *Arabidopsis thaliana* hydroxyproline-rich glycoprotein (HRGP1) mRNA, complete cds [NM_115316.2](http://www.ncbi.nlm.nih.gov/nucleotide/186511057?report=genbank&log$=nucltop&blast_rank=72&RID=4XG75DZN014) | | 0.15 | | 82 | | | Hydroxyproline-rich glycoprotein precursor [*Phaseolus vulgaris*] [AAA87902.1](http://www.ncbi.nlm.nih.gov/protein/727264?report=genbank&log$=prottop&blast_rank=26&RID=50ZA40UM01N) | 2.00E-04 | 68 |
| HSZW1U101A1SZ9 | *Dendrobium crumenatum* putative pectin methylesterase mRNA, partial cds [EU309724.1](http://www.ncbi.nlm.nih.gov/nucleotide/163638043?report=genbank&log$=nucltop&blast_rank=18&RID=4YFW5Z5E014) | | 2.00E-04 | | 94 | | | Conserved hypothetical protein [*Ricinus communis*] [XP_002514143.1](http://www.ncbi.nlm.nih.gov/protein/255546167?report=genbank&log$=prottop&blast_rank=1&RID=4Z04UJ45014) | 0.001 | 55 |
| HSZW1U101A1LPY | *Nicotiana.tabacum* mRNA for extensin  [X71602.1](http://www.ncbi.nlm.nih.gov/nucleotide/296616?report=genbank&log$=nucltop&blast_rank=6&RID=4YDSPYK5014) | | 2.00E-57 | | 90 | | | Predicted protein [*Hordeum vulgare* subsp. *vulgare*] [BAJ99414.1](http://www.ncbi.nlm.nih.gov/protein/326508294?report=genbank&log$=prottop&blast_rank=1&RID=50KNJ21M016) | 1.00E-04 | 43 |
